# Meiotic transmission patterns of additional genomic elements in *Brachionus asplanchnoidis*, a rotifer with intraspecific genome size variation

**DOI:** 10.1038/s41598-022-25566-8

**Published:** 2022-12-03

**Authors:** Julie Blommaert, Claus-Peter Stelzer

**Affiliations:** 1 Research Department for Limnology, University of Innsbruck, Mondsee, Austria; 2 The New Zealand Institute for Plant and Food Research Ltd., Nelson, New Zealand

**Keywords:** Evolutionary ecology, Evolutionary biology

## Abstract

Intraspecific genome size (GS) variation in Eukaryotes is often mediated by additional, nonessential genomic elements. Physically, such additional elements may be represented by supernumerary (B-)chromosomes or by large heterozygous insertions into the regular chromosome set. Here we analyze meiotic transmission patterns of Megabase-sized, independently segregating genomic elements (ISEs) in *Brachionus asplanchnoidis*, a planktonic rotifer that displays an up to two-fold intraspecific GS variation due to variation in size and number of these elements. To gain insights into the meiotic transmission patterns of ISEs, we measured GS distributions of haploid males produced by individual mother clones using flow cytometry and compared these distributions to theoretical distributions expected under a range of scenarios. These scenarios considered transmission biases resembling (meiotic) drive, or cosegregation biases, e.g., if pairs of ISEs preferentially migrated towards the same pole during meiosis. We found that the inferred transmission patterns were diverse and ranged from positive biases (suggesting drive) to negative biases (suggesting drag), depending on rotifer clone and its ISE composition. Additionally, we obtained evidence for a negative cosegregation bias in some of the rotifer clones, i.e., pairs of ISEs exhibited an increased probability of migrating towards opposite poles during meiosis. Strikingly, these transmission and segregation patterns were more similar among members of a genetically homogeneous inbred line than among outbred members of the population. Comparisons between early and late stages of haploid male embryonic development (e.g., young synchronized male eggs vs. hatched males) showed very similar GS distributions, suggesting that transmission biases occur very early in male development, or even during meiosis. Very large genome size was associated with reduced male embryonic survival, suggesting that excessive amounts of ISEs might be detrimental to male fitness. Altogether, our results indicate considerable functional diversity of ISEs in *B. asplanchnoidis*, with consequences on meiotic transmission and embryonic survival.

## Introduction

Many eukaryotes display intraspecific genome size (GS) variation due to varying amounts of non-coding DNA^1–5^. Such GS variation can be mediated by additional genomic elements, which are physically represented either by extra (B-)chromosomes or by large heterozygous insertions into the regular chromosomes. On a DNA sequence level, non-coding DNA can be classified as highly repetitive, e.g. interspersedly repeated transposable elements or tandemly repeated satellite DNA, or as the result of previous duplications of the genome followed by pseudogenization^6^. The long-term gain and loss of such non-coding DNA sequences is thought to be governed by largely neutral evolutionary processes, and their excessive accumulation in some genomes can be explained by genetic drift^7,8^, even though selection might also sometimes play a role^9,10^.

Non-coding DNA can affect organisms in different ways. A large number of studies document correlations between genome size and organismic traits such as cell size^11,12^, body size^13,14^, or developmental rates^15^, sometimes even at the within-population level^13^. Under some circumstances, differential amounts of non-coding DNA might even affect fitness^16^. Furthermore, DNA can have coding-independent effects that operate at lower levels, such as intragenomic selection. For example, (additional) genomic elements might increase their own fitness by increasing their transmission rates to offspring by meiotic drive, sometimes at the expense of their host’s fitness^17–19^. Meiotic drive in this classical sense occurs during the chromosome segregation during the meiotic divisions, even though later stages during gametogenesis can also be affected^20^. Recognizing and disentangling such effects is important for a better understanding of the evolution of eukaryotic genomes, in particular, the evolutionary causes of the large intraspecific genome size variation.

**Figure 1:**
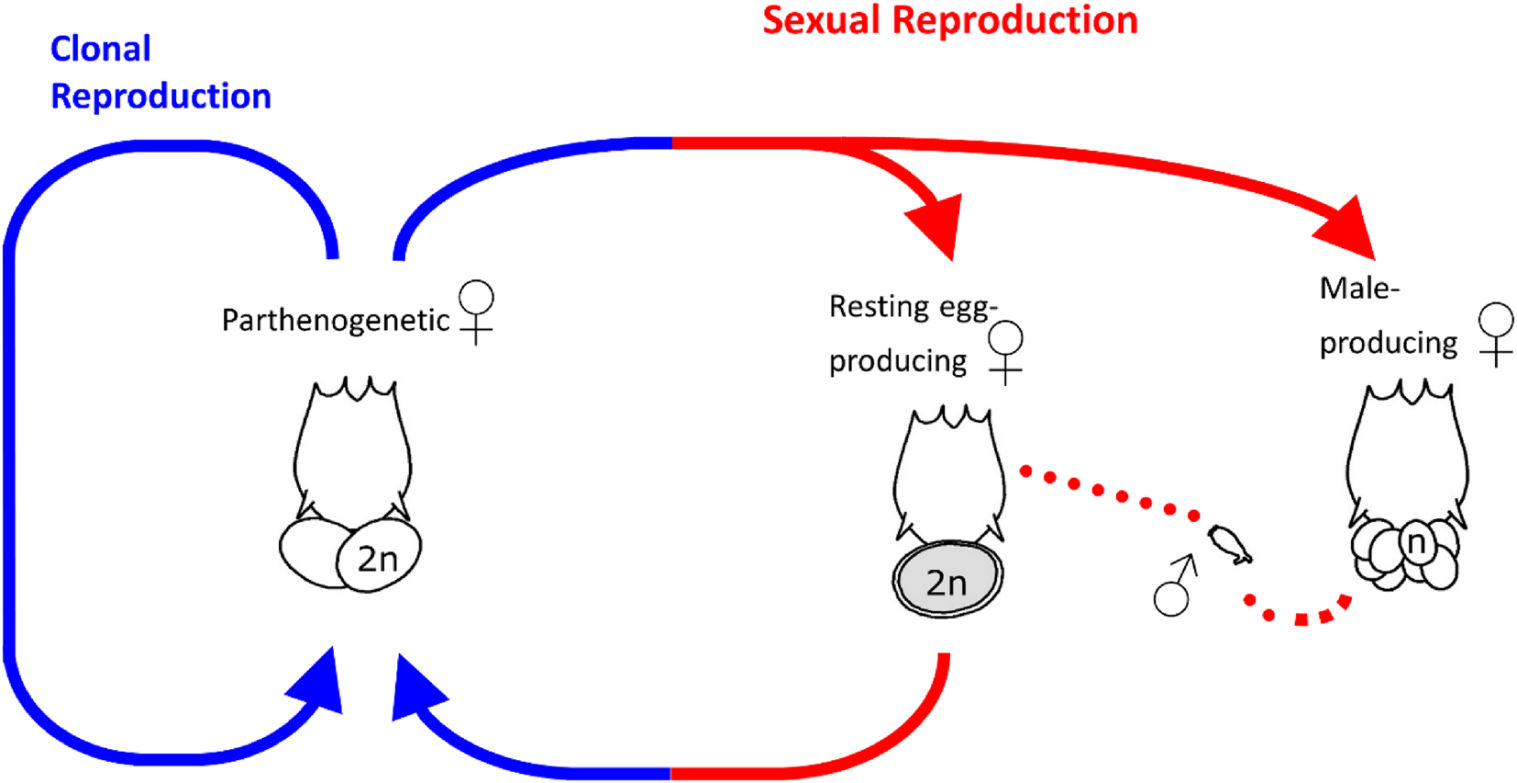
Schematics of rotifer life cycle. Monogonont rotifers are cyclical parthenogens, capable of both ameiotic parthenogenesis and sexual reproduction. The production of sexual females is triggered by *quorum sensing* chemicals, released by the animals themselves at high population density. In contrast to parthenogenetic females, sexual females produce oocytes by meiosis, and give rise to either haploid males or diploid resting eggs, depending on whether they get fertilized by a male^24^.

Here we study meiotic transmission patterns of additional genomic elements in the monogonont rotifer *Brachionus aplanchnoidis.* Individuals of this species can differ by up to almost two-fold in genome size, which is mediated by several Megabase-sized independently segregating genomic elements (ISEs) consisting mainly of tandemly repeated satellite DNA^21^. The genomic data are consistent with a mixture of both B-chromosomes and large insertions to normal chromosomes^21,22^. Individual rotifers and their clonal offspring can be characterized by the number and size of their ISEs and their composition stays constant through hundreds of asexual (mitotic) generations^22^. Occasionally, monogonont rotifers engage in sexual reproduction (Fig. 1), producing sexual females, whose oocytes undergo classical meiosis with two polar bodies formed^23^. Unfertilized haploid eggs develop mitotically into males, and sperm production does not involve any meiotic maturation divisions^24^. By analyzing the genome size distributions of haploid males produced by different mother clones, it has been shown that ISEs segregate in a manner suggesting that they do not pair with each other, nor with any other part of the genome^22^. For instance, a clone containing three ISEs will produce males (and gametes) that might contain either zero, one, two, or three ISEs, corresponding to four different GS classes of the males in this clone. The frequencies of these different GS classes roughly approximated those expected by random segregation. However, previous studies in *B. asplanchnoidis* did not resolve different steps during meiotic transmission, so they were not designed to detect meiotic drive or subsequent changes in meiotic transmission, and they also did not test whether there were subtle deviations from completely independent segregation.

**Figure 2:**
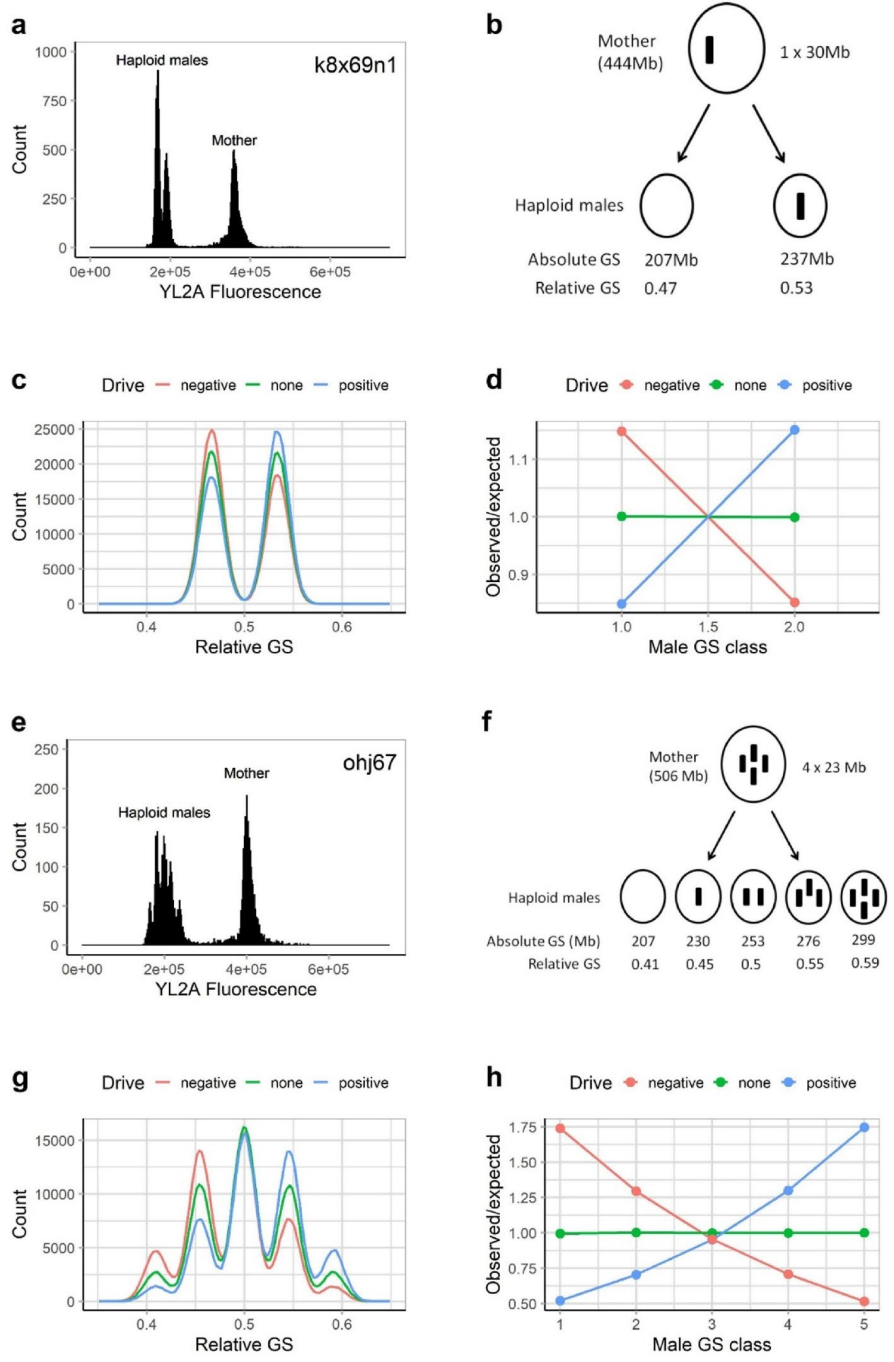
Principle of inferring meiotic transmission patterns from the genome size distributions of haploid rotifer males. The first four panels (**a**–**d)** show a rotifer clone with one ISE (i.e., two corresponding male GS classes). The last four panels (**e**–**h)** show a clone with four ISEs (i.e., five corresponding male GS classes). **a**, **e** Example of flow cytometry data. **b**, **f** Conceptual model of ISE meiotic segregation. **c**, **g** Theoretically predicted GS distributions of males (relative to the female GS) under meiotic drive, meiotic drag, or in the absence of meiotic drive. **d**, **h** Theoretically predicted O/E ratios (observed vs. expected frequencies of different male GS classes) under drive, drag, or on absence of drive. O/E values of > 1 indicate over-representation of a GS class (relative to the frequency expected from unbiased transmission).

In the present study, we test for meiotic transmission biases of ISEs. If meiotic transmission would be completely unbiased, the frequencies of haploid oocytes, or males, with different numbers of ISEs should be identical to those expected by random segregation. For example, a mother with two ISEs should produce males with zero, one, or two ISEs (hence, three male GS classes), which have relative frequencies of 0.25, 0.5, and 0.25, respectively. However, if ISEs avoid segregating into polar bodies due to meiotic drive^17,20,25^, one would expect to see an increase in the relative frequency of male GS classes with two ISEs, compared to those with no ISE . By contrast, if ISEs are preferentially sequestered into polar bodies due to meiotic drag ^7,26^, the GS class with two ISEs should be underrepresented. Our experimental approach for detecting meiotic transmission biases relies on measuring (by flow-cytometry) the observed relative frequencies of each male GS class and comparing these to their relative frequencies expected under unbiased transmission (Fig. 2). To allow for clear comparisons, the main output variable in these analyses is the observed/expected ratio (O/E-ratio), i.e., the observed frequency divided by the expected relative frequency for each GS class. If there were no transmission biases, O/E-ratios across all GS classes should equal one. In contrast, O/E-ratios larger than one indicate overrepresentation of a certain GS class, and if O/E ratios increase or decrease with genome size, this indicates drive or drag at a meiotic or postmeiotic stage (Fig. 2d,h).

We implemented these ideas in a mathematical model that contains the two parameters, *transmission bias* and *cosegregation bias*. Values for *transmission bias* may range from − 1 to 1 in our model. For instance, a value of 0.1 denotes a 10% increase in probability that an ISE segregates towards the egg pole (this is equivalent to a transmission rate of 0.55 for this ISE, i.e. mild meiotic drive). Concerning the second parameter, cosegregation bias, a positive value means that pairs of ISEs have an increased probability of being sequestered towards the same pole (irrespective of whether this is the egg pole or polar body pole), while a negative bias favors migration towards opposite poles. Please note that a cosegregation bias value of − 1 (i.e., 100% probability that ISEs migrate towards opposite poles) resembles the default segregation pattern of regular chromosomes. By estimating the *transmission bias* and *cosegregation bias* parameter for each rotifer clone, we tried to infer and compare general meiotic transmission patterns across clones, even if they contained different numbers and types of ISEs.

Transmission biases may not only arise during meiosis, as described above but also during later stages of male embryonic development. For instance, they might be caused by differences in the survival of embryos, or due to differences in the fitness of hatched males containing different numbers of ISEs. To address these potential sources of variation, we compared the transmission biases in relatively young, synchronized male eggs, older eggs accumulating in growing cultures, and hatched males. Finally, to address the question of whether a high number of ISEs affects male embryonic survival in general, we estimated and compared hatching rates of (haploid) male eggs and (diploid) female eggs in 19 rotifer clones of different genome sizes (which is highly correlated with the number and size of ISEs in the genome^22^).

Our results suggested that the ISEs in *B. asplanchnoidis* exhibit diverse meiotic segregation patterns: In some rotifer clones, *transmission bias* was positive, while the ISEs of other clones showed negative *transmission bias* (indicative of drag). Furthermore, we obtained evidence for a negative *cosegregation bias* in some clones, i.e., pairs of ISEs showed an increased probability to segregate towards opposite poles. Overall, these transmission patterns seemed to be determined early in the haploid life cycle, probably at or shortly after meiosis, since early and late stages of male embryonic development showed very similar GS distributions. Finally, we found that very large genome size (i.e., a large numbers of ISEs) was associated with reduced male embryonic survival.

## Methods

### Rotifer culture

Rotifer clonal populations were maintained as in Riss et al.^3^. Briefly, a clonal population of rotifers arises from a single asexual female, originally hatched individually from a resting egg. Since *B. asplanchnoidis* rotifers reproduce asexually, all members of a single clone are genetically identical, but different clones from the same population are not. Here, we focused on various clones and crosses of a population from Obere Halbjochlacke (OHJ, Austria) which have been previously described (see Supplementary Table 1 in^22^). Rotifers were cultured in F/2 medium^27^ at 16 ppt salinity and fed *Tetraselmis suecica* algae at ad libitum concentration (500–1000 cells µl^−1^). Cultures were continually illuminated with daylight LED lamps (SunStrip, Econlux) at 30–40 µmol quanta m^−2^ s^−1^ for rotifers and 200 µmol quanta m^−2^ s^−1^ for algae. Rotifer cultures were grown until male eggs and hatchlings were present and then harvested for further experiments (as below). Clonal stock cultures were kept at 18 °C and were reinoculated once per week by transferring 20 asexual females to fresh culture medium provided in 20 ml Petri dishes. The clones used in this study and the genome size of the females (2C) can be found in Table S1.

### Egg isolation and hatching rate determination

Rotifer cultures were filtered through a 63 µm nylon sieve and rinsed with F/2 medium to remove algae. To isolate rotifer eggs, they were removed from females by passing the egg-bearing adults through a 0.5 µm needle and collected into F/2 medium. In this study, we had two main treatments concerning the eggs. *Synchronized eggs* are eggs that had been produced in a time interval of up to 8 h, which is considerably less than the development time of female eggs or male eggs (18 h or 21.5 h, see^13^). Synchronization was accomplished by stripping eggs from the same females two times, first to obtain females without eggs and a second time (after up to 8 h) to isolate the newly produced eggs. *Accumulated eggs* are eggs of unknown age, isolated from females that were growing in clonal culture for at least a week. If there are eggs that did not complete development but are still attached to the female, these eggs should be enriched compared to the synchronized-egg treatment. To determine hatching rates, eggs (female or male) were placed in individual wells of 48-well plates with 0.5 mL sterile F/2 medium. Eggs were incubated at 21 °C and monitored over 48 h, and hatching rates were recorded at the end of the 48 h.

### Flow cytometry measurements

Genome size was estimated by flow cytometry with propidium iodide staining as per^22^. Female genome sizes were previously determined, and for each clone, the female was used as an internal standard. Rotifer culture was as above, with rotifers isolated once males and male eggs were present in high numbers. Females were harvested using 63 µm sieves and starved overnight in 11ppt salt medium before staining. Males, who do not feed, did not require starvation. For each sample, up to 200 hatchling males or male eggs were isolated into 11ppt medium, and 30–80 starved females were added depending on the genome size of the female in that clone. Rotifers were then washed twice with the 11ppt medium, and transferred to a citrate buffer (3.4 mM Trisodium citrate dihydrate, Nonidet P40 at 0.1% v/v, 1.5 mM Sperminetetrahydrochloride, 0.5 mM Trishydroxymethylaminomethane, pH 7.6), and homogenized in 750ul stock solution with 30 strokes of the “tight” pestle of a 1 mL Dounce tissue homogenizer on ice. The samples were then filtered through a 40 µm mesh nylon sieve, and digested with 100 μl of 0.021% Trypsin for 10 min at 37 °C. This digestion was then incubated at 37 °C for 10 min after the addition of 75 µl of 0.25% trypsin inhibitor (this solution also included 0.05% RNAse A). Then, propidium iodide stain was added to a concentration of 50 µg mL^−1^, and incubated overnight on ice in the dark. An Attune NxT acoustic-focusing flow cytometer (ThermoFisher) was used with an excitation wavelength of 561 nm and a custom-made 590–650 nm bandpass filter (yellow, YL-2) to detect propidium iodide fluorescence. The flow cytometry data (Supplementary file 1) were analyzed using FlowJo software (v 10.0.7r2, FlowJo LLC.). To exclude doublets (nuclei that pass the detector too close together, thus being recorded as a single “event”), we employed YL2-A *vs*. YL2-H gating. The coefficients of variance (CVs) of female peaks ranged between 1.7 and 3.95%, and measurements were excluded from further analyses if CVs were above 3.5%. YL2-A fluorescence data were then exported in CSV-format for further analyses in R (see below).

To identify and quantify male GS classes, we used finite mixture models as implemented in the R package mixsmsn v1.1.10^28^ on the YL2-A fluorescence data (Supplementary Fig. 2). Briefly, finite mixture models are clustering algorithms that consider an overall population (here, the GS distribution of males produced by a rotifer clone) as the sum of a finite set of subpopulations. Instead of using hard thresholds, i.e., exclusive ranges YL2-A values for each GS class, finite mixture models estimate the proportion of each GS class based on probability distributions. We used skewed t-distributions to approximate these subpopulations, which have been previously used in flow cytometric studies (e.g.^29^). Within the mixsmsn package, we used the function smsn.search with the main parameters set to g.min = 3, g.max = 8, family = “Skew.t”, and the optimization parameters set to criterion = “bic”, error = 0.00001, and iter.max = 1000. Note that the parameter g.max = 8 implies that up to 8 subpopulations may be fitted and included in the final model—if this improves the fit according to the Bayesian information criterion. Even though this number is higher than the number of male GS classes, we found that increasing g.max to 8 often improved the resolution of the GS classes, especially in datasets with higher background fluorescence. Since this overfitting sometimes resulted in more than one subpopulation for a certain GS class, we calculated the proportion of this GS class as the sum of its subpopulations, and its mean YL2-A value as the weighted mean of the fitted subpopulations. Subpopulations that were fitted to background noise were excluded from further analysis, and proportions of the GS classes were recalculated so that they summed to one. The main output of this analysis was the relative frequency of each male GS class, e.g., 0.54 and 0.46 in the case of two GS classes (one ISE).

### Mathematical model

To gain insights into the mechanisms affecting the meiotic transmission of ISEs, we developed a theoretical model with two parameters influencing their segregation, *transmission bias* and *cosegregation bias*. Our model is loosely based on the one used by Stelzer et al.^22^, which predicts the genome size of males (or females) within a rotifer clone based on the number and size of the ISEs in that clone and assuming their completely unbiased and independent segregation. These assumptions are relaxed in our new model. The new parameter *transmission bias* specifies an increased chance that an ISE is represented in the gametes/males. For example, a *transmission bias* value of 0.1 indicates that the chance of an ISE segregating towards the egg pole is increased by 10% (hence, it has a probability of 0.55 to migrate towards the egg pole). Thus, *transmission bias* is a measure of drive or, in the case of negative values, drag. The second parameter *cosegregation bias* applies only to genomes with two or more ISEs and specifies an increased chance of two ISEs segregating into the same gamete/male after meiosis. Positive values of *cosegregation bias* indicate that two ISEs tend to associate and migrate towards the same pole, while negative values indicate that two ISEs have an increased probability of segregating to opposite poles, i.e., they tend to behave according to Mendel’s law. The main model output is the predicted relative frequencies of the different male genome size classes. For example, in a clone with two ISEs, three different male genome size classes are possible: males without an ISE, males with one ISE, and males with both ISEs. Without any biases or interactions, their expected relative frequencies are 0.25:0.5:0.25, while nonzero values for *transmission bias* and/or *cosegregation bias* will alter these frequencies. The code of the model was written in R v4.1.2^30^ and is available in Supplementary file 2.

To estimate *transmission bias* and *cosegregation bias* for ISEs in different rotifer clones, we calculated the theoretical male genome size distributions across all combinations of the two parameters ranging from − 0.95 to 0.95. Next, we compared these theoretical distributions to the empirical distributions in different rotifer clones, which were obtained using flow cytometry. The best-fitting combinations were identified by the lowest residual mean squared error. Overall, we analyzed clones with numbers of ISEs ranging from 1 to 5 (hence 2–6 male genome size classes per clone). Some of these data were re-analyzed from an earlier study^22^ while others were obtained within the present study (clones ohj67 and k8x69n1). The total set included 11 clones of an inbred laboratory line (ohj7i3 in^22^) and 12 outbred clones, which were either sampled from the natural population or crossed offspring of unrelated individuals.

### Statistical analysis

All statistical analyses were carried out in R v4.1.2^30^. In clone ohj67, the effects of male GS class and stage (accumulated eggs, hatched males) on the observed/expected ratio were investigated using the *lm* function. For clone k8x69n1, which has only two male GS classes, we tested for transmission bias via testing for effects of treatment (synchronized eggs, accumulated eggs, hatched males) on the proportion of offspring in the first male GS class (the GS class with no ISE) using repeated G-tests for goodness-of-fit in the R-package RVAideMemoire v0.9-81-2^31^. To prepare the data for these tests, we reconstructed the number of males in each GS class based on the number of males used in each sample and the proportion of the first GS class as it was previously determined by flow cytometry and finite mixture modelling (see above). In addition, we used repeated G-tests of goodness-of-fit to test if the proportion of offspring in the first male GS class deviated from the expected ratio of 0.5. The effects of sex (male or female) and genome size on egg hatching rates were analyzed using the function *glm* with binomial error structure. All plots were produced using ggplot2 v 3.1.1 and cowplot v1.1.1^32,33^.

## Results

**Figure 3:**
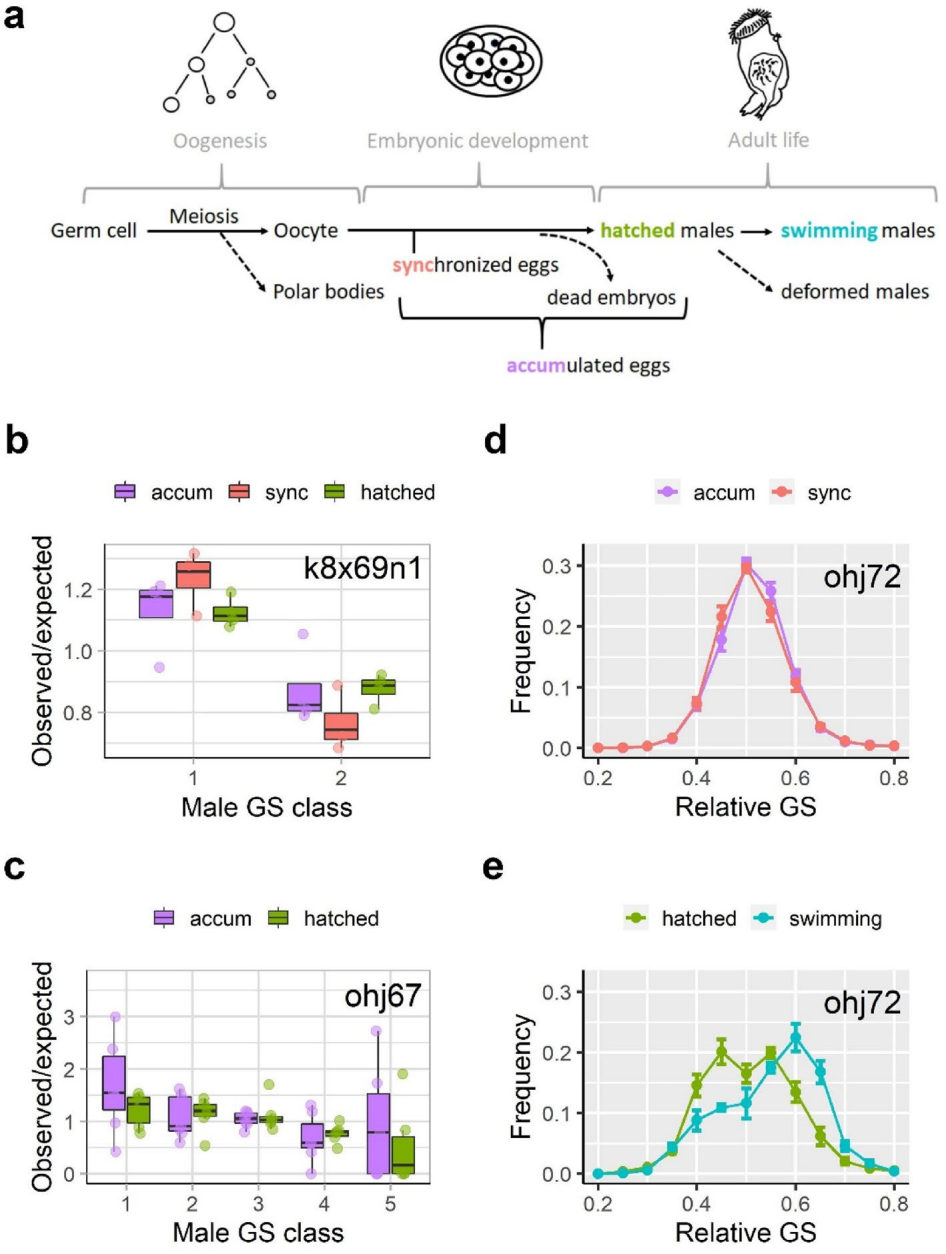
Meiotic transmission biases during different stages of male embryonic development. (**a**) Schematic of male embryonic development and the stages that were distinguished: Synchronized eggs (*sync*) are haploid male eggs with a known age of < 6–8 h, which is shorter than the embryonic development time (~ 20 h). Accumulated eggs (*accum*) are eggs of unknown age sampled from sexual females of a rotifer population, which should contain a larger fraction of dead embryos, compared to *sync*. Hatched males (*hatched*) are the fraction of embryos that completed development. In one rotifer clone, ohj72, we analyzed an additional category of males (*swimming*) as the fraction of hatchlings that was viable, as opposed to deformed males in the same clone, which were unable to swim properly. (**b**) Analysis of k8x69 (Female GS: 444 Mb), a clone with one ISE and two male genome size classes. (**c**) Analysis of ohj67 (Female GS: 506 Mb), a clone with four ISEs and five male genome size classes. (**d**, **e**) Analysis of ohj72, a clone with an unknown, but presumably very high number (> 10) of ISEs. In this clone individual male GS classes could not be resolved using flow cytometry. Thus, relative GS instead of discrete GS classes is given.

To quantify meiotic transmission of ISEs, we estimated genome size distributions of different stages during male embryonic development (Fig. 3a): in relatively young synchronized eggs (i.e., closer to meiosis), in eggs accumulating in a growing culture, and hatched males. In a clone with one ISE (k8x69n1, Fig. 3b), there was no significant effect of stage (Heterogeneity G-test: G = 17.44, *df* = 11, *p* = 0.09552), yet, the smaller male GS class (the one without an ISE) was significantly more abundant than the expected frequency of 0.5 (Pooled G-test: G = 59.697, *df* = 1, *p* = 1.106e−14). In a clone with four ISEs (ohj67, Fig. 3c), the situation was analogous in that the smallest male GS classes were significantly more abundant (Table S2) than expected under the assumption of unbiased transmission (Observed/expected ratios > 1), while for the largest GS classes the pattern was reversed. In this same clone, there was no notable difference between accumulated eggs and hatched males. In a third clone with a more complex complement of ISEs, discrete male GS classes cannot be resolved by flow cytometry^22^, thus we observed an extremely broad GS distribution of the males produced by this clone, spanning from ca. 30% to 70% of female genome size (ohj72, Fig. 3d,e). While there was no difference in the genome size distributions of synchronized *vs.* accumulated eggs, the genome size of hatched males was biased towards extreme genome sizes, both lower and higher than the mean genome size, resulting in an overall broader GS distribution. In this same clone, we detected a high number of apparently deformed males (Fig. S2), which were partially to completely unable to swim. Non-deformed males of this clone (i.e. “swimming” males in Fig. 3e) had larger genome sizes on average, and thus contained a higher number of ISEs.

To assess the behavior of our theoretical model, we examined a wide range of parameter space, including situations where one of the two parameters was held at zero (Fig. S3), and situations where both parameters vary together (Fig. S4). As expected, positive values of the model parameter *transmission bias* are associated with monotonically increasing overrepresentation of the GS classes with higher numbers of ISEs, manifesting as increasing O/E ratios, while negative values of *transmission bias* show the opposite pattern (Fig. S3a,c,e). In contrast, the model parameter *cosegregation bias* affects the O/E patterns in a non-monotonic manner. At negative values of *cosegregation bias*, extreme GS classes (i.e., GS classes with all or no ISE) tend to be underrepresented (O/E ratios < 1), while at positive values of *cosegregation bias*, the pattern is reversed (Fig. S3b,d,f). For example, in modelled clones with three male GS classes (2 ISEs) and a *cosegregation bias* of − 0.3 (i.e., by 30% increased probability that a pair of ISEs will segregate into opposite poles) the two extreme GS classes (0 and 2 ISEs) are both 0.7-fold underrepresented while the intermediate GS class (1 ISE) is 1.3-fold overrepresented (Fig. S3b). In the two other theoretical cases, four GS classes (3 ISEs) and five GS classes (4 ISEs), *cosegregation bias* had a similar effect of increasing or decreasing O/E ratios of extreme GS classes (Fig. S3d,f). Overall, the effects of *transmission bias* and *cogegregation bias* are additive, as can be seen in Fig. S4. For instance, at positive *transmission bias* and negative *cosegregation bias*, O/E ratios overall tend to increase with GS-class (number of ISEs), but the extreme GS classes are still underrepresented relative to the intermediate ones.

**Figure 4:**
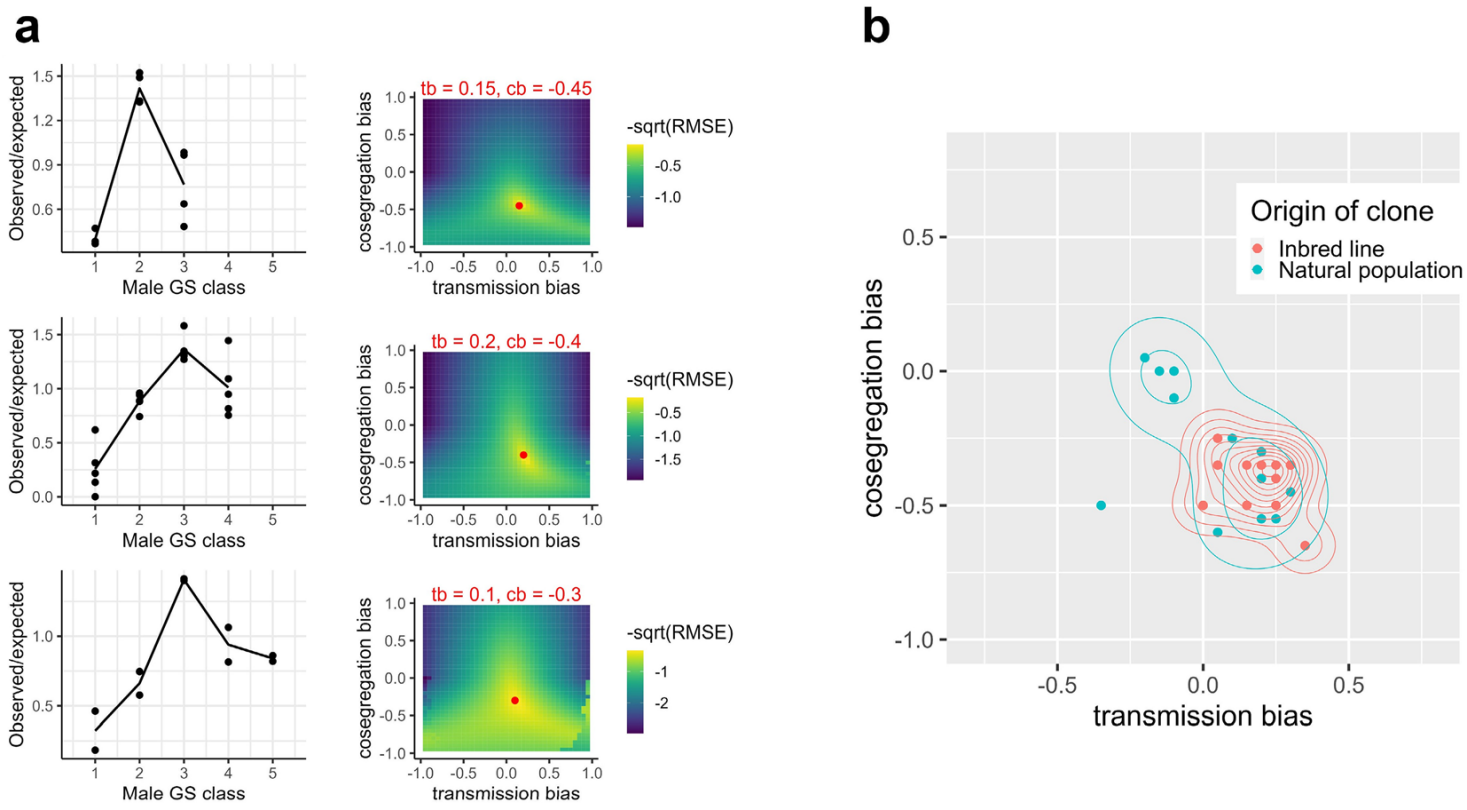
Estimation of meiotic transmission parameters. (**a**) Least-squares estimation of the optimal parameter values for *transmission bias* (tb) and *cosegregation bias* (cb) based on empirical GS data (hatched males) from an inbred laboratory line. Left: Empirical data (Clonal means indicated by dots and means across clones indicated by lines). Right: Model fitted to the means (across clones) of each genome size class. The best combination of *transmission bias* and *cosegregation bias* with the lowest residual mean square error (RMSE) is indicated by a red dot. (**b**) The same fitting procedure was applied to genome size data of hatched males of 11 rotifer clones from the inbred line and 12 outbred clones. Dots correspond to the least-squares estimations of the optimal parameter values for the different clones in Supplementary file 3. Contours are 2D kernel density estimations for inbred and outbred clones (function: *geom_density_2d* of the R-package *ggplot2*).

To test for *transmission bias* and *cosegregation bias* in *B. asplanchnoidis*, we compared the predictions of our theoretical model to the empirical distributions of male GS classes. In an inbred laboratory line, we examined clones with three, four, and five GS classes, respectively (hence, two, three, and four ISEs). Overall, meiotic transmission was biased, with observed/expected ratios increasing with genome size and the intermediate GS classes 2 and 3 being overrepresented (Fig. 4a). The best-fitting models had values for *transmission bias* ranging from 0.1 to 0.2 and *cosegregation bias* ranging from − 0.3 to − 0.45. Applying the same fitting procedure to data of individual clones, both from the inbred line and several outbred natural clones, shows that the inbred line centers around slightly positive values for *transmission bias* (around 0.15) and moderately negative values for *cosegregation bias* (around − 0.35) while the natural clones encompass at least one additional cluster with slightly negative values of *transmission bias* (around − 0.1) and *cosegregation bias* values of around zero (Supplementary file 3, Fig. 4b). Hence, even though we examined a similar number of clones (11 inbred vs. 12 outbred), meiotic transmission patterns of outbred clones appear to be more diverse.

**Figure 5:**
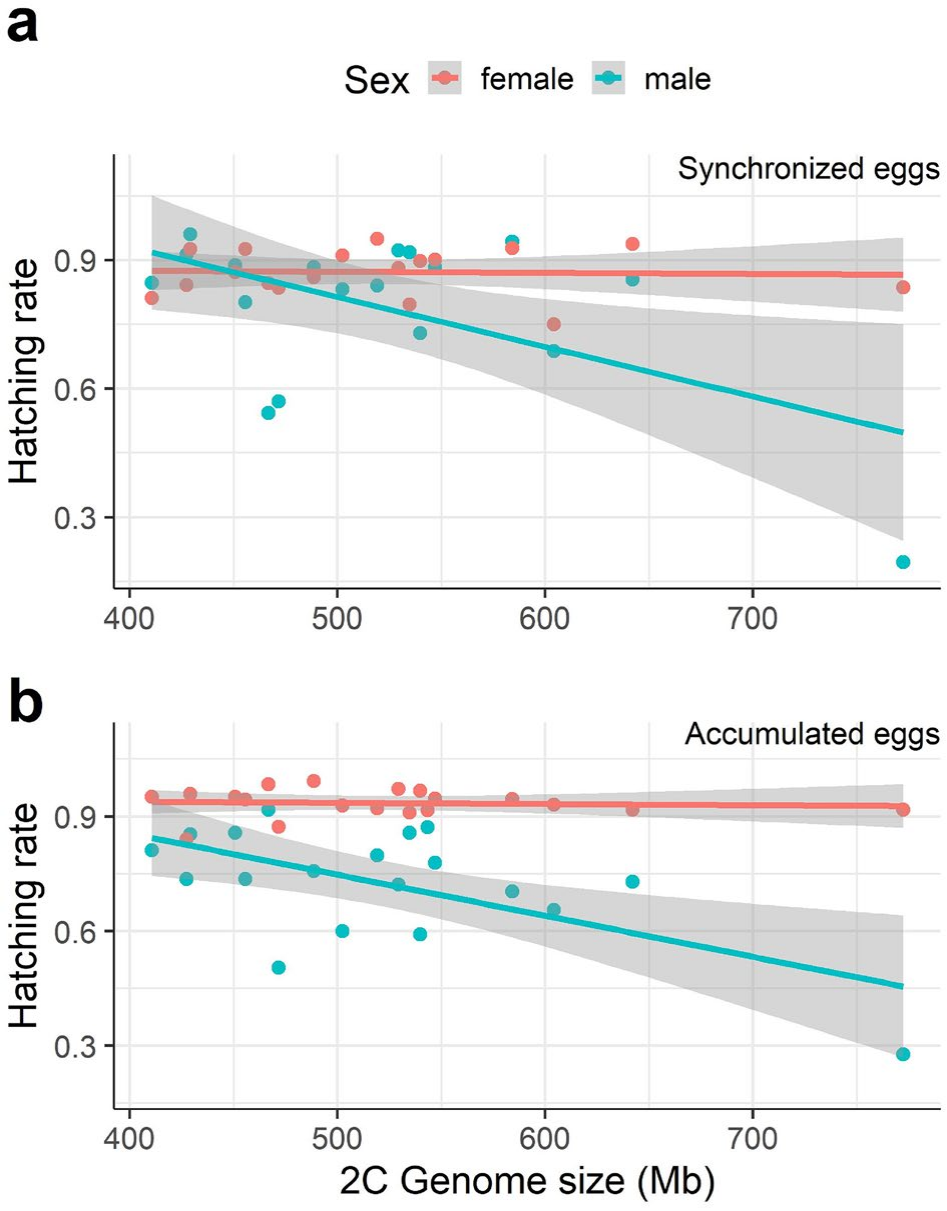
Relationship between hatching rate and genome size in male *vs.* female eggs. (**a**) In synchronized eggs (i.e., eggs aged < 8 h), hatching rates declined with genome size in males, but not in females, as indicated by a significant negative GS-by-sex interaction in the GLM (*p* < 0.001, Table S3). (**b**) In accumulated eggs (i.e., eggs of unknown age, collected from females in a growing culture), hatching rates were lower in males, and the interaction between genome size and sex was significant (*p* < 0.001, Table S4). Each colored symbol represents a rotifer clone (*n* = 19 clones), shaded areas indicate the 95% confidence intervals. Source data of this figure are provided in Supplementary file 4.

To investigate factors influencing male embryonic survival, we measured hatching rates (= successful embryonic development) of male *vs*. female eggs across a wide range of genome sizes in natural OHJ-clones. Overall, male hatching rate was negatively affected by genome size while female hatching rate was not (Fig. 5, Tables S3, S4). We also found that male hatching rates were significantly lower than female hatching rates, especially in the accumulated egg treatment (Fig. 5b, Table S4). This strengthens our initial assumption that accumulated eggs contain a large fraction of embryos with stalled development.

## Discussion

Additional genomic elements, called ISEs in *B. asplanchnoidis*, are the main driver of intraspecific and intrapopulation genome size variation^22^. Consisting predominantly of satellite DNA^21^, ISEs are characterized by a GC-content very different from the rest of the genome (≥ 37% GC in ISEs *vs*. 25% GC in the core genome). In the present study, we show that ISEs can experience drive, with *transmission bias* values of 0.1–0.15 (corresponding to transmission ratios of 0.55–0.6) to drag (i.e., *transmission bias* ~ − 0.1; transmission ratio: ~ 0.45). Compared to other animal systems, these values seem relatively low^34^, although there might be a publication bias towards high values in the literature. In addition, drag, i.e., the tendency of ISEs to be sequestered into polar bodies during meiosis, might be less often reported (but see^26^). Overall, the observed diversity of transmission patterns complements our earlier findings, which have also identified a diverse range of sizes of ISEs in the *B. asplanchnoidis* natural population, with some elements being 34 Mb in size while others are 21 Mb or smaller^22^.

We also found that in some clones, ISEs exhibit an interesting transmission pattern that we term “repulsion” (two ISEs in the same genome showing increased probability to move towards opposite poles during meiosis), which was indicated by negative values of the model parameter *cosegregation bias*. In other words, a pair of ISEs behaves to some extent like a regular chromosome pair rather than segregating completely independently. Repulsion was mainly observed in our inbred lineage, but it could also be detected in some of the natural isolates (Fig. 4b). Whether this cytological interpretation accurately reflects the true meiotic behavior of the ISEs remains to be investigated. Such experiments would require microscopic observations of condensed chromosomes during meiosis. Unfortunately, even visualizing mitotocally dividing chromosomes has proven extremely challenging in this species^22^. Nevertheless in our dataset, negative values for the parameter *cosegregation bias* were required to account for the characteristic overrepresentation of the intermediate male GS classes observed in flow-cytometry data, both in inbred and outbred clones (Fig. 4a, Supplementary file 3).

According to our results, these transmission biases are determined relatively early in male embryonic development, possibly already in meiosis. This was suggested by the very similar transmission biases in several stages of male embryonic development in two clones with a known number of ISEs (k8x69n1 and ohj67, Fig. 3b,c). By contrast, in ohj72, a clone with an extremely large genome size (2C: 792 Mb) and an unknown number of ISEs, there could also be post-meiotic transmission biases, such that male embryos that contain fewer ISEs fail to develop properly and hatch with deformities (Fig. 3e), and are therefore unable to swim properly. Regarding its effects on the phenotype, this would be equivalent to a meiotic drive of the “sperm killing” segregation distortion type, in which propagules not carrying the driving element are selectively eliminated^20^. Even though this is an interesting hypothesis and warranting future investigation, we have so far observed male deformities just in this one clone, so the association with large genome size could be incidental.

Meiotic drive elements operate at a level below the individual, thus they may spread despite reducing an organism’s fitness. In the present study, male hatching rate was negatively affected by genome size, a proxy for the number of ISEs^22^. Although we did not find a negative effect of genome size on female hatching rate, a recent study found that female embryos of large GS clones develop more slowly and, in some cases, large GS clones experienced slightly reduced asexual population growth^13^. However, whether this phenotype is detrimental might depend on the ecological setting, as in some situations a “large and slow” phenotype might in fact be advantageous^35^.

In natural populations, the spread of meiotic drivers can be limited or even prevented by suppressor genes in the host^34^. Thus, an alternative explanation to our results on the diversity of transmission patterns might be that the genomic background, rather than the type of ISE, is diverse and determines whether ISEs will experience drive or drag. Testing this hypothesis would require isolating different types of ISEs into inbred lines, and then crossing them into controlled ISE-free genomic backgrounds. Ideally, such work would include genome sequencing to track genomic variants that are involved in drive suppression. As long as such data are missing, however, the most parsimonious explanation remains that the ISEs themselves are the cause for the diversity of transmission patterns.

In conclusion, we have shown that supernumerary genomic elements in *B. asplanchnoidis* can experience various transmission biases, from drive to drag, and that there are additional interactions among elements (e.g., “repulsion”) that may affect how two elements in the same host genome are distributed among the gametes. How such processes affect genome size variation in this animal system and how they affect long-term genome size evolution remains to be investigated. Theoretically, supernumerary genomic elements, being present either as B-chromosomes or as large insertions to the regular chromosome set could rapidly drive up genome size if not countered by drive-suppressing genomic mechanisms and/or large fitness costs. Future work should disentangle the roles of these two counter-acting mechanisms in maintaining the impressive, almost two-fold intraspecific genome size variation of *B. asplanchnoidis*.

## Supplementary Information

Supplementary Information 1.

Supplementary Information 2.

Supplementary Information 3.

Supplementary Information 4.

## Data Availability

All data generated or analysed during this study are included in this published article and its supplementary information files.
