# Supplementary material for: Meiotic transmission patterns of additional genomic elements in Brachionus asplanchnoidis, a rotifer with intraspecific genome size variation
Source: Sci Rep. 2022 Dec 3;12:20900. doi: 10.1038/s41598-022-25566-8 (PMC9719556; doi:10.1038/s41598-022-25566-8)
Supplement: Supplementary file 1 — Supplementary Information 1. [file 41598_2022_25566_MOESM1_ESM.docx]

Supplementary Information for:

**Meiotic transmission patterns of additional genomic elements in *Brachionus asplanchnoidis*, a rotifer with intraspecific genome size variation**

Julie Blommaert ^1,2^ & Claus-Peter Stelzer ^1,^*

^1^ Research Department for Limnology, University of Innsbruck, Mondsee, Austria

^2^ The New Zealand Institute for Plant & Food Research Limited Nelson, New Zealand

* Corresponding author: claus-peter.stelzer@uibk.ac.at

This file contains:

- Figures S1-4
- Tables S1-4
- References for supplementary information

Other supplementary data provided with this publication, but not contained in this document:

1. Raw flow cytometry data files (DOI:10.1038/s41598-022-25566-8)
2. R-code of the mathematical model (*Suppl_File_2_maleGSmodel.R*)
3. Fitting model to male GS from different rotifer clones (*Suppl_File_3_modelfits.doc*)
4. Count data of hatching rate experiment (*Suppl_file_4_HRdata.csv*)


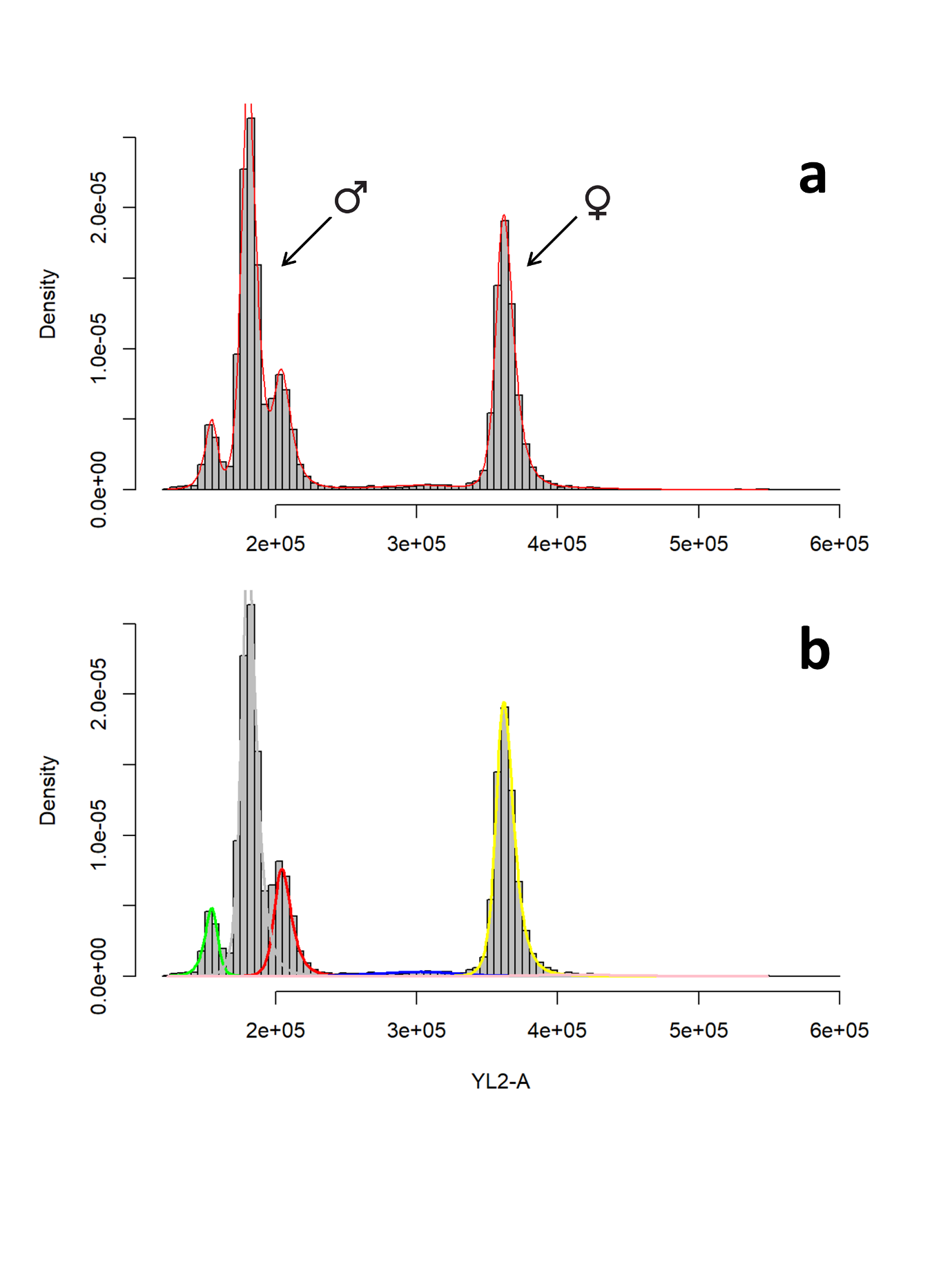


**Figure S1. Example of a finite mixture model fit.** This example shows ik7, a clone with 3 male genome size classes, corresponding to males with zero, one, and two ISEs. **a** Full model (red line) and flow cytometry data (bar histogram). **b** Model components (curves in green, grey, red, and yellow) and flow cytometry data. Model fitting was done using the *smsn.search* function within the R-package *mixsmsn* (Prates et al. 2013).


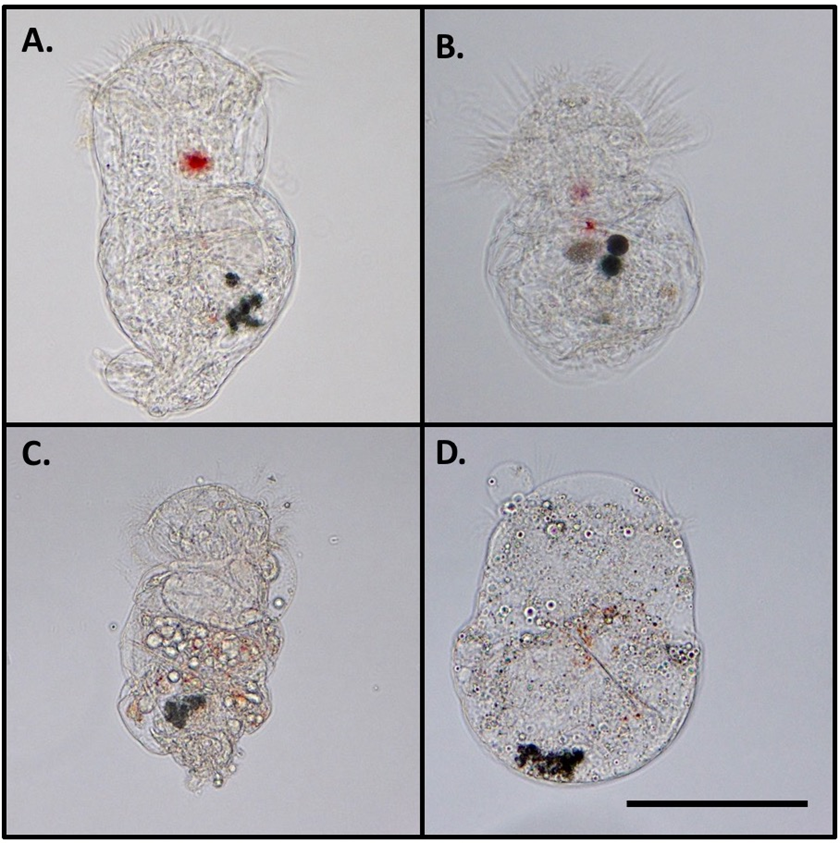


**Figure S2.** **The range of morphologies observed in hatched males of clone ohj72.** The rotifers which were deformed, but still swimming (**a**, **b**), had a curved shape to their body and swam slowly and mostly in circles. Their internal morphology seemed to be largely unaffected (e.g., eye spot, presence of sperm). The more severely-affected males that did not swim anymore (**c**, **d**) still had a corona, and seemed to have sperm, but the eyespot was more diffuse through the body, rather than a focal spot just posterior of the corona. Males of the categories **a** and **b** were regarded as “swimming” (c.f., **Fig. 3 a, e**). Scale bar indicates 100 µm.


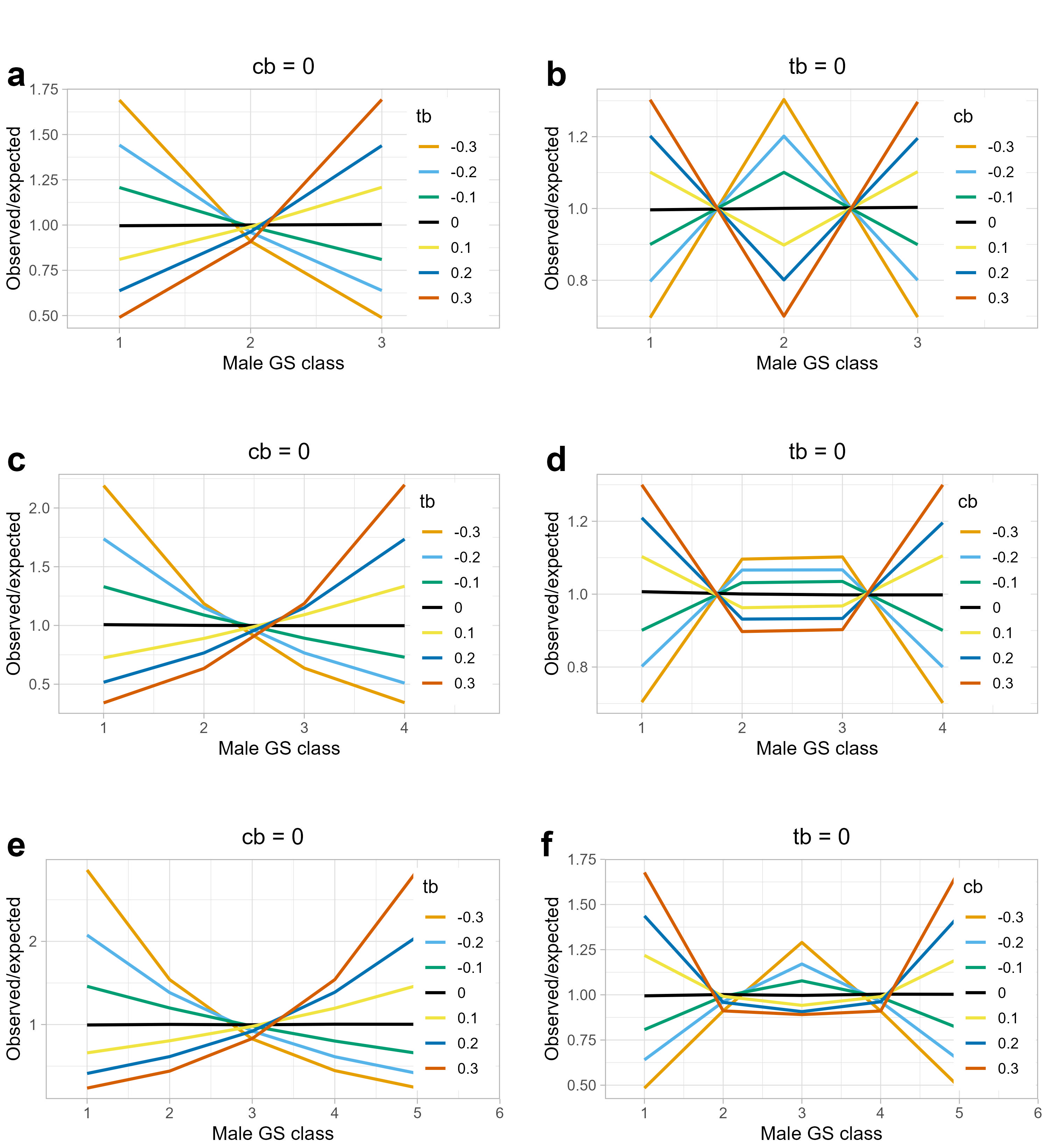


**Figure S3. Examples of predictions of the meiotic transmission model.** One of the two parameters, *transmission bias* (tb) or *cosegregation bias* (cb), is held at zero while the other is varied from -0.3 to 0.3. Predictions for three different rotifer clone types: **a & b** Clones with three male genome size classes, mediated by 0-2 equally sized independently segregating elements (ISEs), **c & d** Clones with four male GS classes, mediated by 0-3 ISEs, **e & f** Clones with five male GS classes, mediated by 0-4 ISEs.


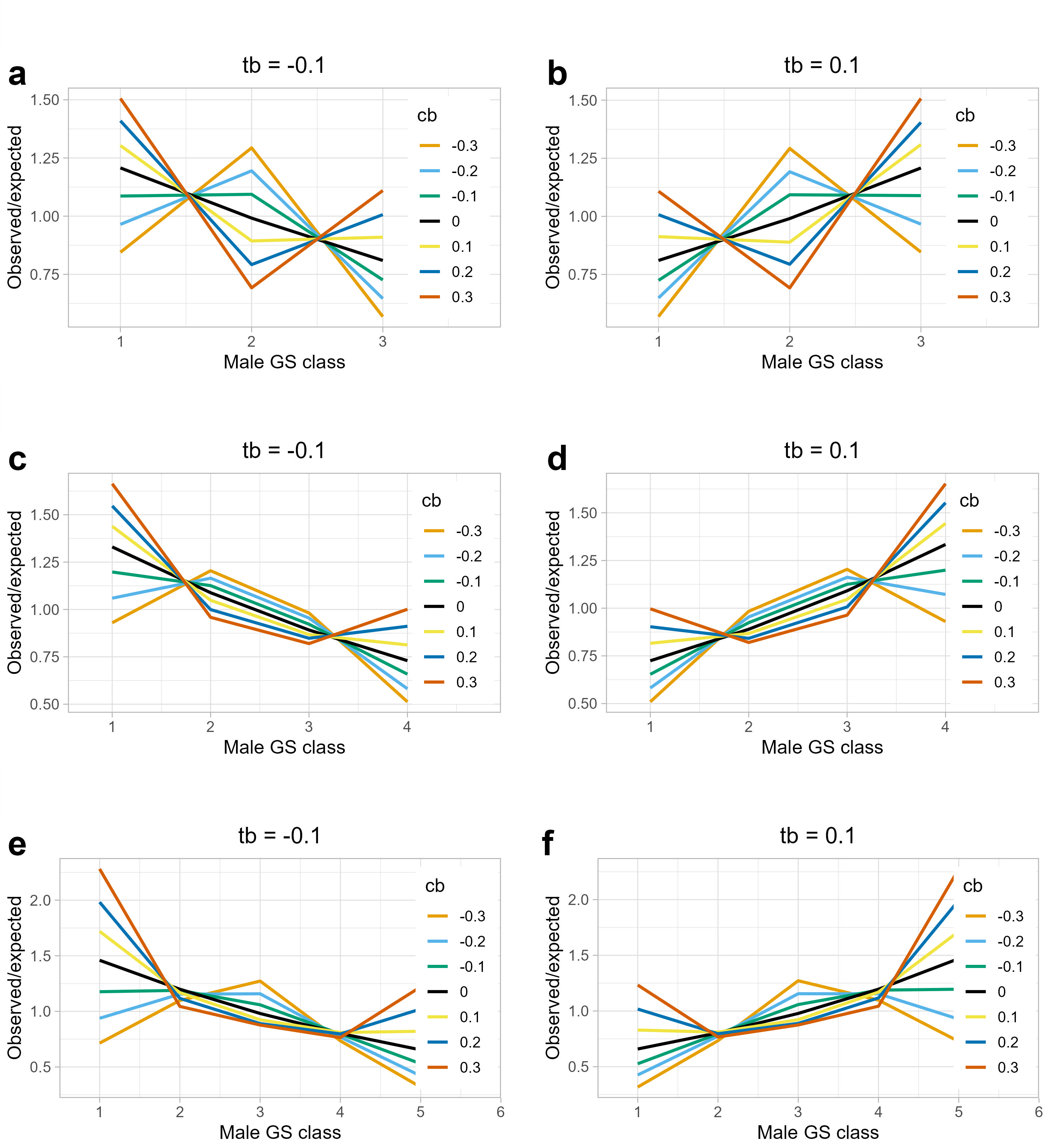


**Figure S4. Examples of predictions of the meiotic transmission model for positive (0.1) and negative values (-0.1) of the parameter *transmission bias* (tb).** Predictions for three different rotifer clone types: **a & b** Clones with three male genome size classes, mediated by 0-2 equally sized independently segregating elements (ISEs), **c & d** Clones with four male GS classes, mediated by 0-3 ISEs, **e & f** Clones with five male GS classes, mediated by 0-4 ISEs.

**Table S1. Rotifer clones used in this study**

| **Clone** | **Origin** | **Genome size (2C, Mb) ^1^** | **# ISEs** |
| --- | --- | --- | --- |
| ohj7 | outbred | 532 | 3 |
| ohj13 | outbred | 536 | n.d. |
| ohj64 | outbred | 480 | n.d. |
| ohj66 | outbred | 546 | n.d. |
| ohj67 | outbred | 548 | 4 |
| ohj72 | outbred | 798 | >6 |
| ohj76 | outbred | 474 | 2 |
| ohj80 | outbred | 480 | 2 |
| ohj85 | outbred | 460 | 2 |
| ohj90 | outbred | 486 | 2 |
| ohj96 | outbred | 492 | 2 |
| ohj98 | outbred | 504 | 3 |
| ohj103 | outbred | 464 | 2 |
| ohj105 | outbred | 520 | 4 |
| k(13x110)x(13x66)n12 | outbred | 638 | n.d. |
| k(13x110)x(13x66)n13 | outbred | 600 | n.d. |
| k109x13n2 | outbred | 524 | 3 |
| k109x67n1 | outbred | 528 | n.d. |
| k110x109n1 | outbred | 498 | 4 |
| k13x109n1 | outbred | 474 | 1 |
| k13x110n1 | outbred | 584 | n.d. |
| k13x110n2 | outbred | 492 | 1 |
| k22x73n1 | outbred | 400 | n.d. |
| k7x107n1 | outbred | 526 | n.d. |
| k8x69n1 | outbred | 466 | 1 |
| k8x82n1 | outbred | 430 | n.d. |
| k8x86n1 | outbred | 428 | n.d. |
| k93x22n1 | outbred | 456 | 1 |
| k93x69n1 | outbred | 476 | 2 |
| ik1 | inbred | 500 | 2 |
| ik2 | inbred | 500 | 2 |
| ik3 | inbred | 498 | 2 |
| ik4 | inbred | 526 | 3 |
| ik5 | inbred | 540 | 3 |
| ik6 | inbred | 524 | 3 |
| ik7 | inbred | 510 | 2 |
| ohj7i2n11 | inbred | 574 | 4 |
| ohj7i3n2 | inbred | 560 | 4 |
| ohj7i3n7 | inbred | 536 | 3 |
| ohj7i3n8 | inbred | 522 | 3 |
| ^1^ Data from Stelzer et al. (2019); n.d.: not determined. | | | |

**Table S2. Effects of male GS class and stage (accumulated eggs, hatched males) on transmission bias (O/E ratio) in clone ohj67.**

|  |  |  |  |  |  |  |
| --- | --- | --- | --- | --- | --- | --- |
|  | **Df** | **Sum Sq.** | **Mean Sq.** | ***F* value** | **Pr(>*F*)** |  |
| GS class | 1 | 4.501 | 4.501 | 14.10 | 0.00039 | *** |
| stage | 1 | 0.327 | 0.327 | 1.03 | 0.315 |  |
| GS class X stage | 1 | 0.008 | 0.008 | 0.02 | 0.877 |  |
| Residuals | 61 | 19.478 | 0.319 |  |  |  |
|  |  |  |  |  |  |  |

**Table S3. Relationship between hatching rate and genome size in synchronized eggs.** GLM model and analysis of deviance. This table corresponds to the data displayed in **Fig. 5**.

|  |  |  |  |  |  |
| --- | --- | --- | --- | --- | --- |
|  | **Estimate** | **Std. Error** | **z value** | **Pr(>\|z\|)** |  |
| (Intercept) | 1.9380 | 0.4103 | 4.72 | 2.31E-06 | *** |
| sex | 3.1660 | 0.5234 | 6.05 | 1.46E-09 | *** |
| genome size | 0.0001 | 0.0008 | 0.10 | 0.93 |  |
| sex X genome size | -0.0072 | 0.0010 | -7.15 | 8.91E-13 | *** |
|  |  |  |  |  |  |
|  |  |  |  |  |  |
|  |  |  |  |  |  |
|  | **df** | **Deviance** | **df (Deviance)** | **Pr(>Chi)** |  |
| NULL |  |  | 35 |  |  |
| sex | 1 | 44.099 | 34 | 3.12E-08 | *** |
| genome size | 1 | 85.327 | 33 | 2.20E-16 | *** |
| sex X genome size | 1 | 56.131 | 32 | 6.78E-11 | *** |
|  |  |  |  |  |  |

**Table S4. Relationship between hatching rate and genome size in accumulated eggs.** GLM model and analysis of deviance. This table corresponds to the data displayed in **Fig. 5**.

|  |  |  |  |  |  |
| --- | --- | --- | --- | --- | --- |
|  | **Estimate** | **Std. Error** | **z value** | **Pr(>\|z\|)** |  |
| (Intercept) | 2.2238 | 0.4477 | 4.97 | 6.79E-07 | *** |
| sex | 0.9575 | 0.5139 | 1.86 | 0.06 | . |
| genome size | 0.0006 | 0.0009 | 0.71 | 0.48 |  |
| sex X genome size | -0.0049 | 0.0010 | -4.99 | 5.99E-07 | *** |
|  |  |  |  |  |  |
|  |  |  |  |  |  |
|  |  |  |  |  |  |
|  | **df** | **Deviance** | **df (Deviance)** | **Pr(>Chi)** |  |
| NULL |  |  | 37 |  |  |
| sex | 1 | 480.77 | 36 | 2.20E-16 | *** |
| genome size | 1 | 56.25 | 35 | 6.38E-14 | *** |
| sex X genome size | 1 | 27.19 | 34 | 1.84E-07 | *** |
|  |  |  |  |  |  |

**References for supplementary information**

Prates, M. O., Lachos, V. H. & Barbosa Cabral, C. R. mixsmsn: Fitting Finite Mixture of Scale Mixture of Skew-Normal Distributions. Journal of Statistical Software 54, 1 - 20, doi:10.18637/jss.v054.i12 (2013).

Stelzer, C. P., Pichler, M., Stadler, P., Hatheuer, A. & Riss, S. Within-Population Genome Size Variation is Mediated by Multiple Genomic Elements That Segregate Independently during Meiosis. Genome Biol Evol 11, 3424-3435 (2019).
