# Supplementary material for: Meiotic transmission patterns of additional genomic elements in Brachionus asplanchnoidis, a rotifer with intraspecific genome size variation
Source: Sci Rep. 2022 Dec 3;12:20900. doi: 10.1038/s41598-022-25566-8 (PMC9719556; doi:10.1038/s41598-022-25566-8)

## Supplementary data 3

The following graphs show least-squares estimations of the optimal parameter values for *transmission bias* (tb) and *cosegregation bias* (cb) for different rotifer clones.


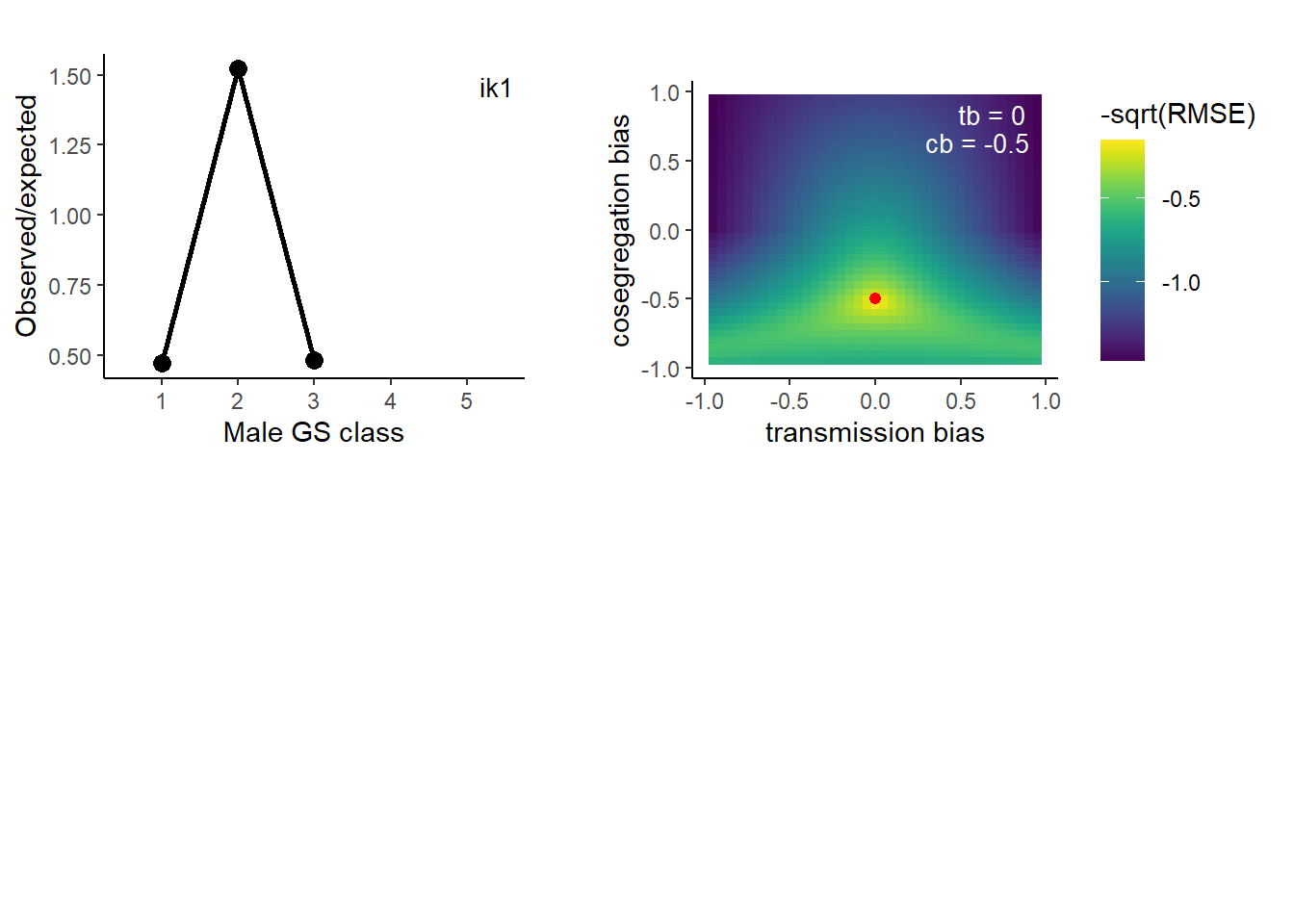

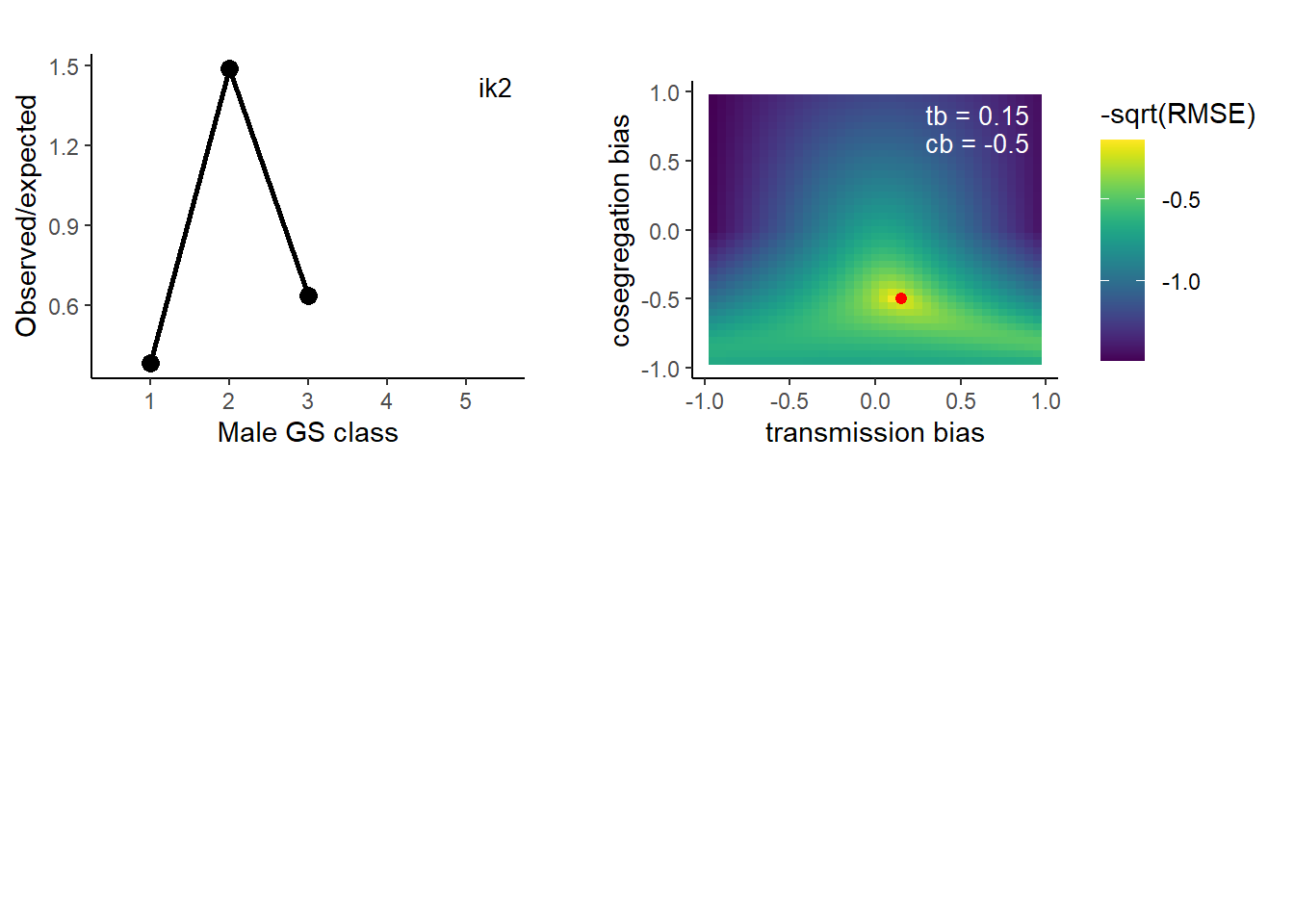

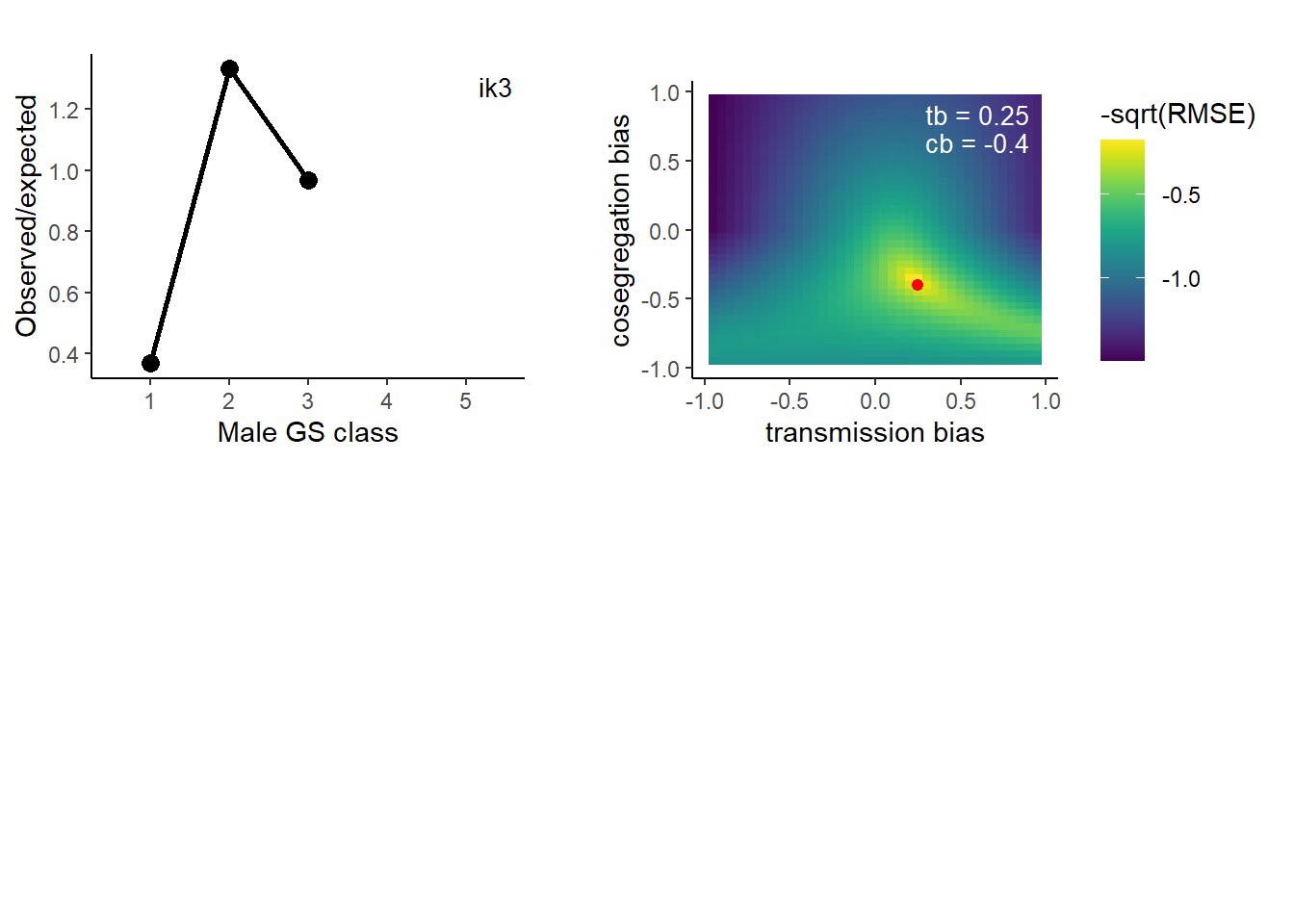

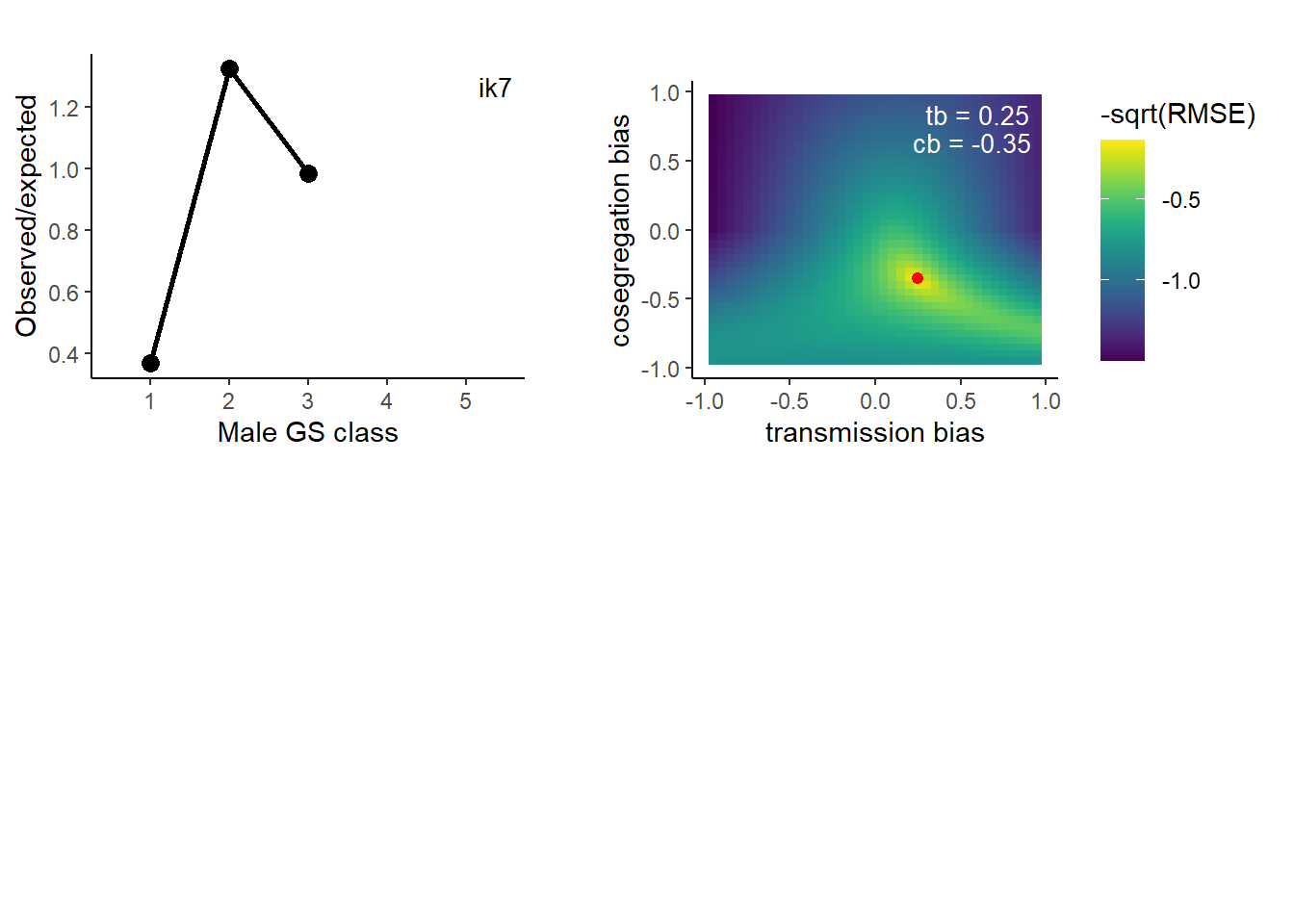

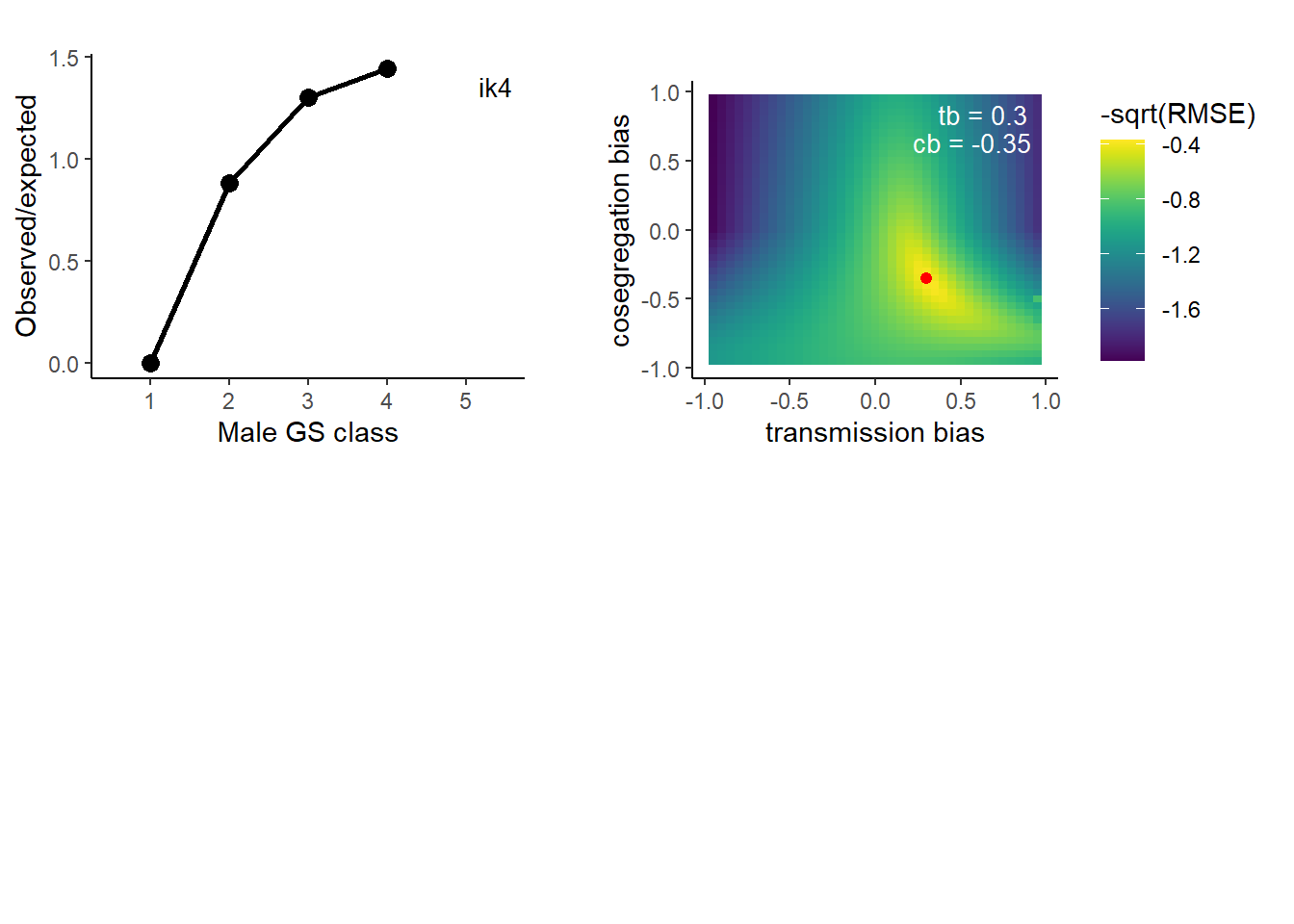

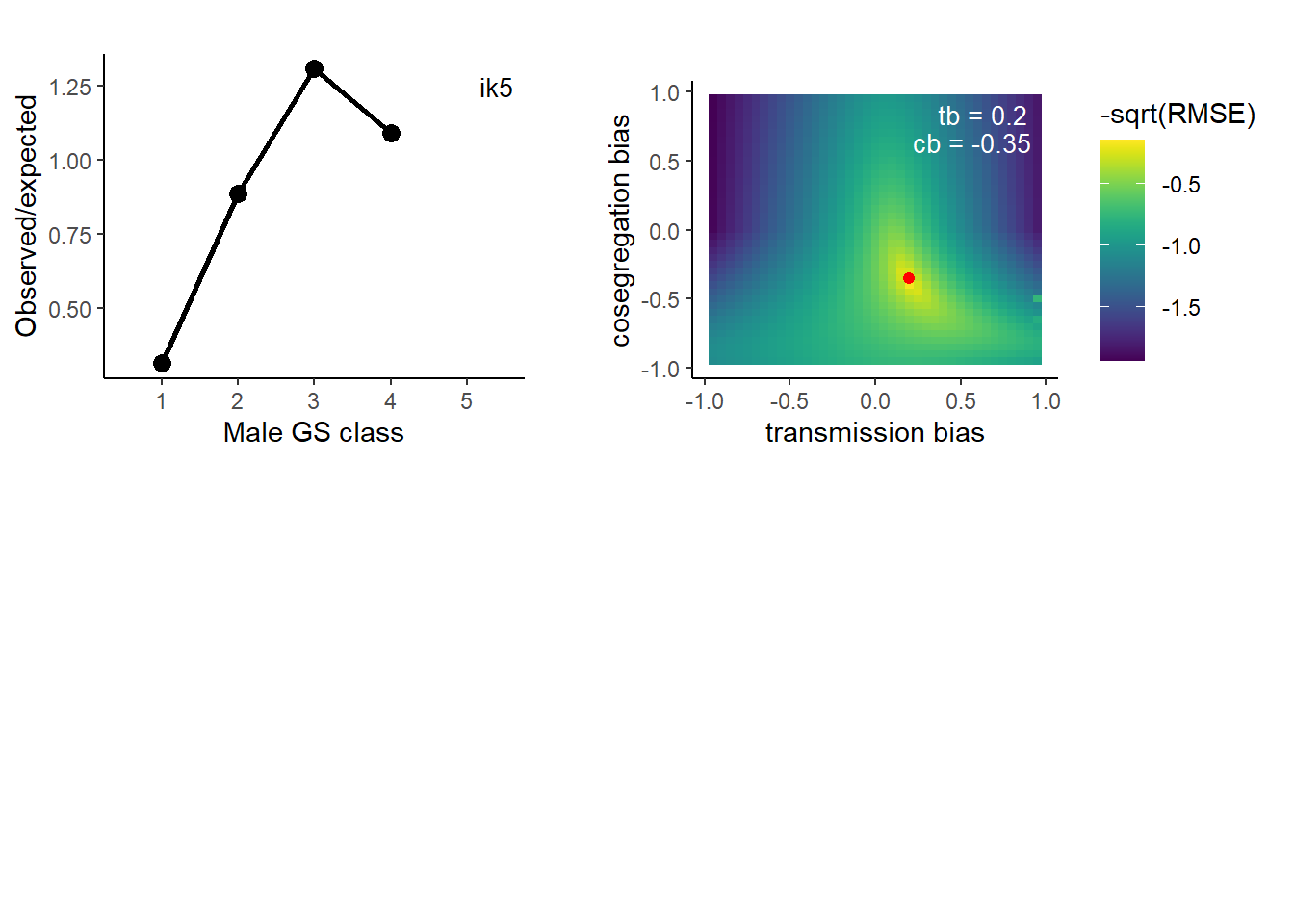

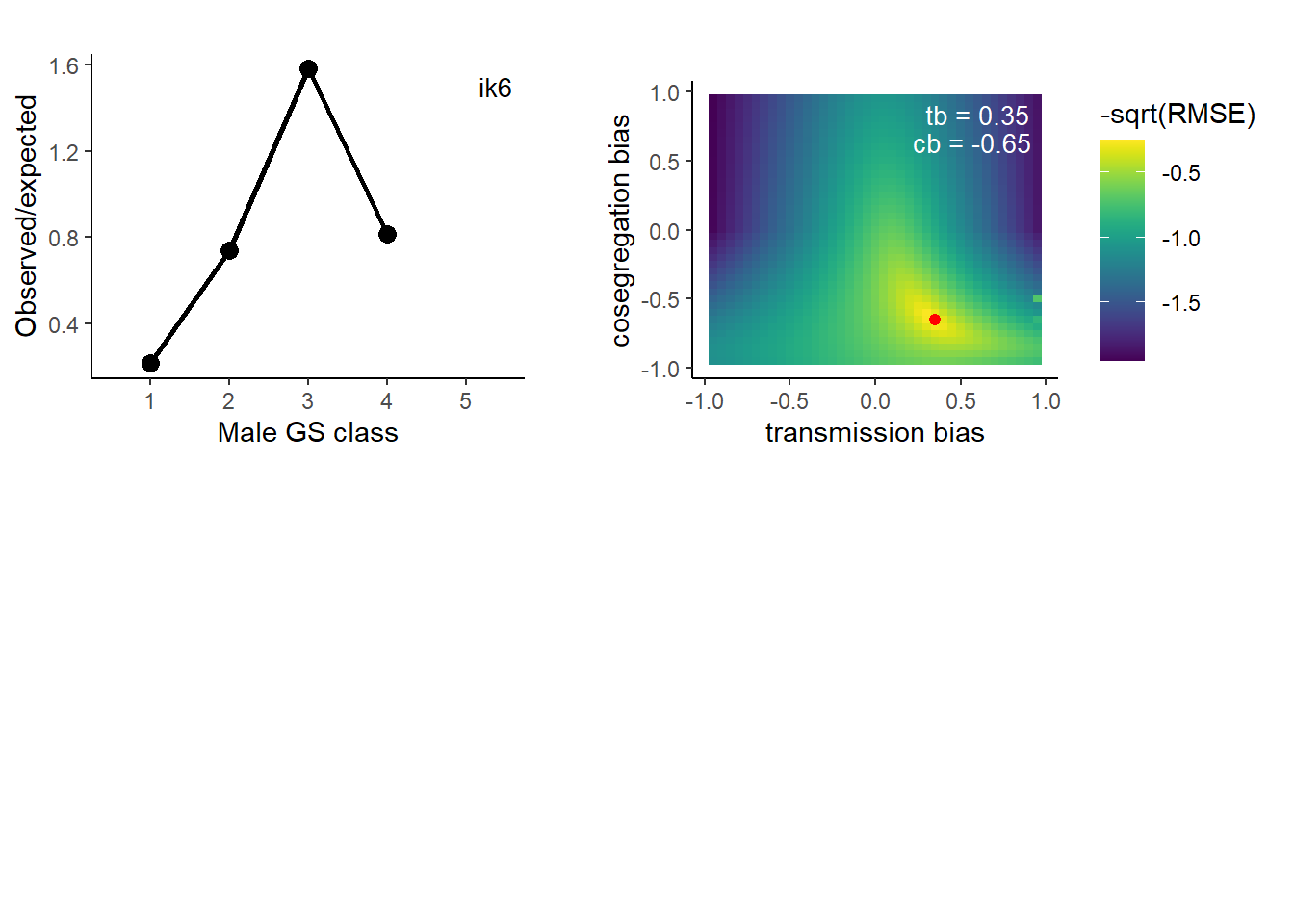

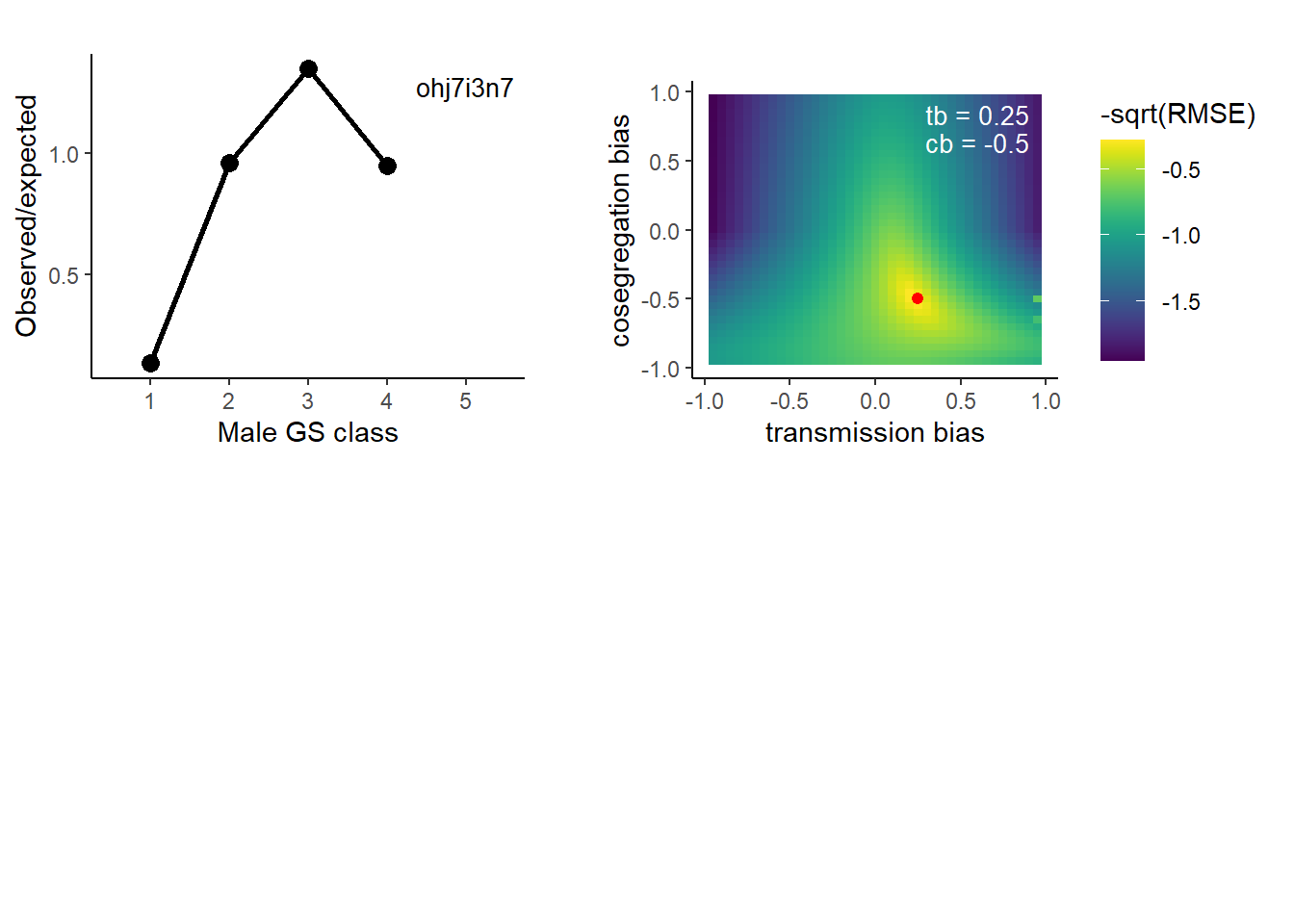

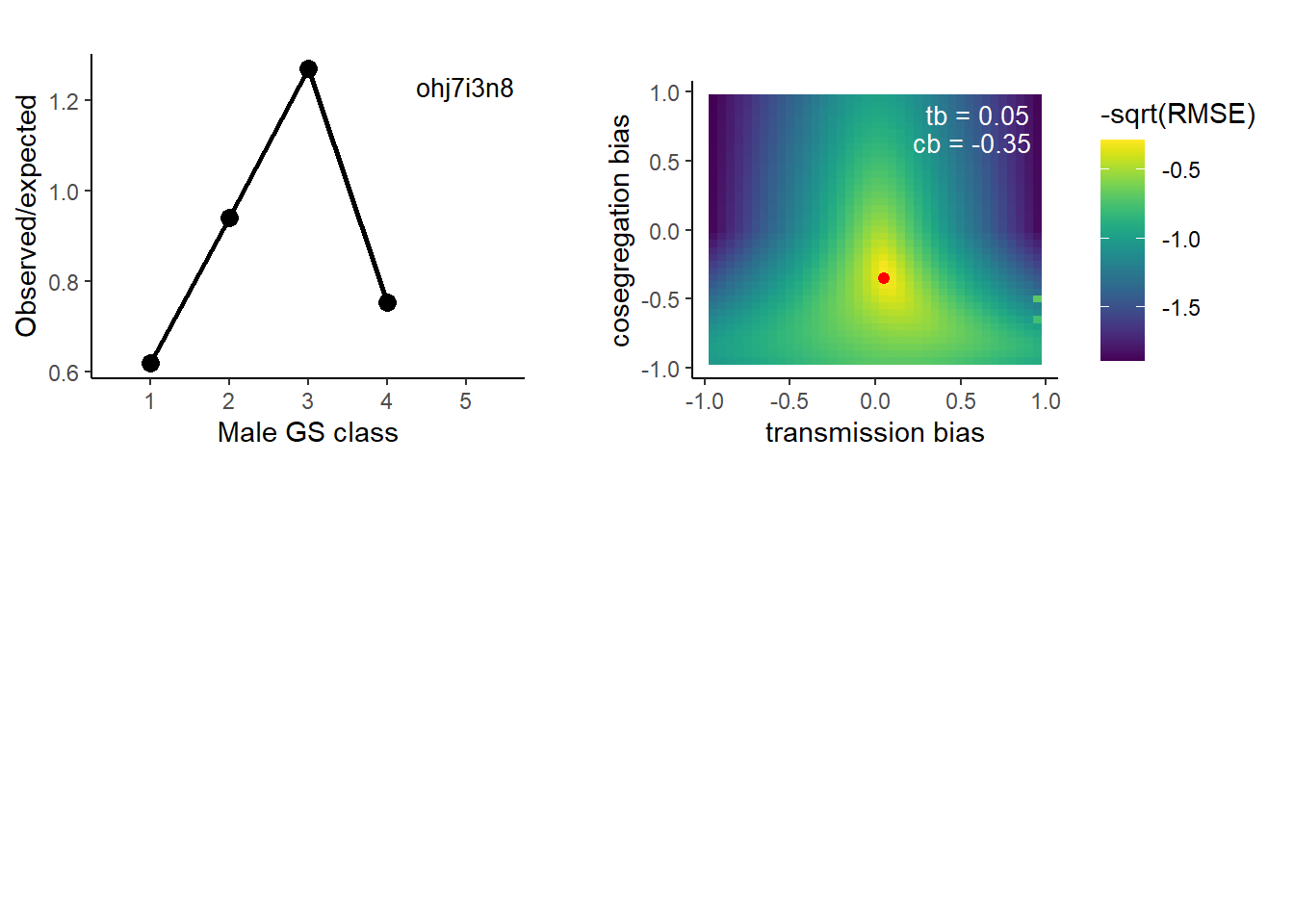

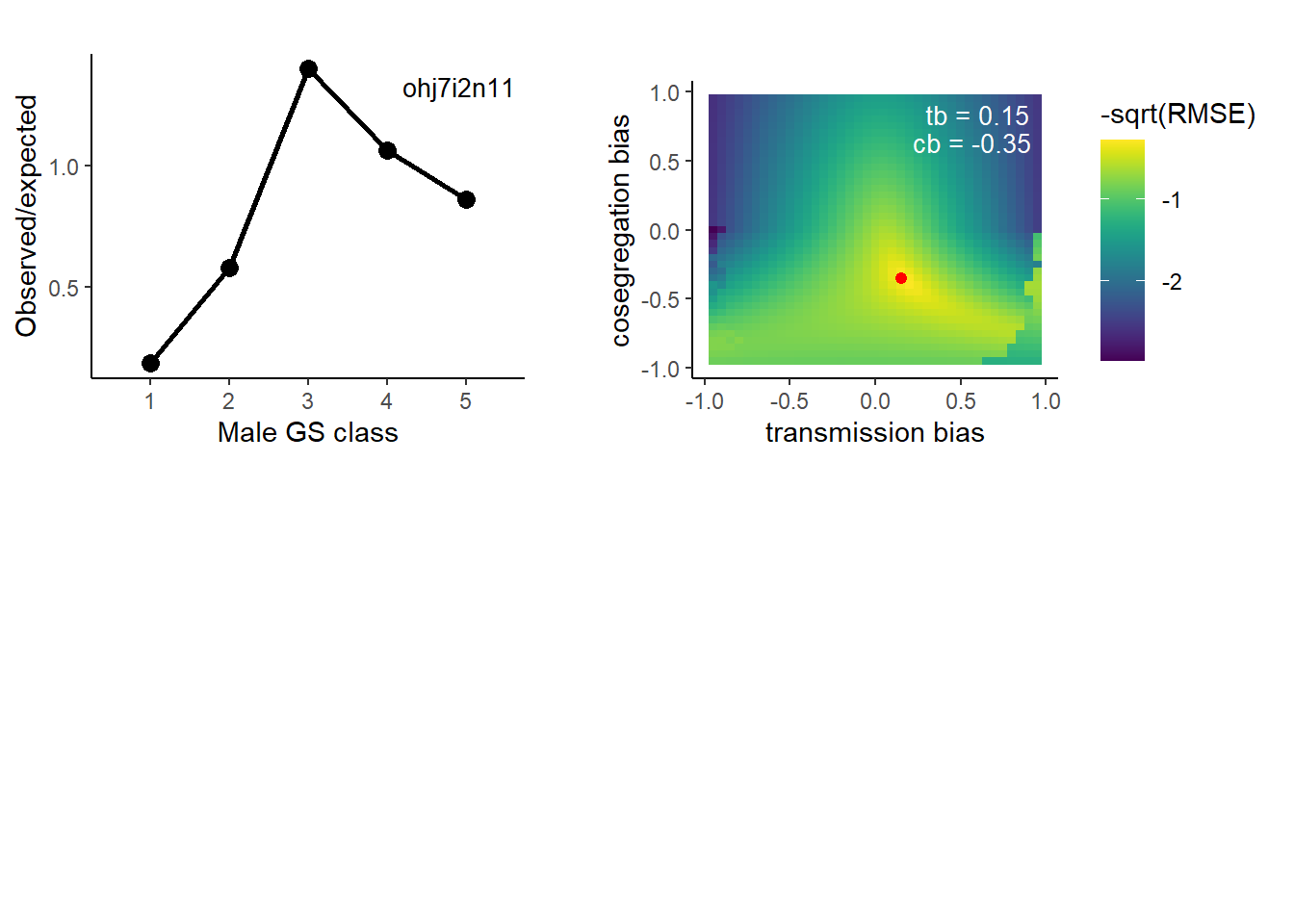

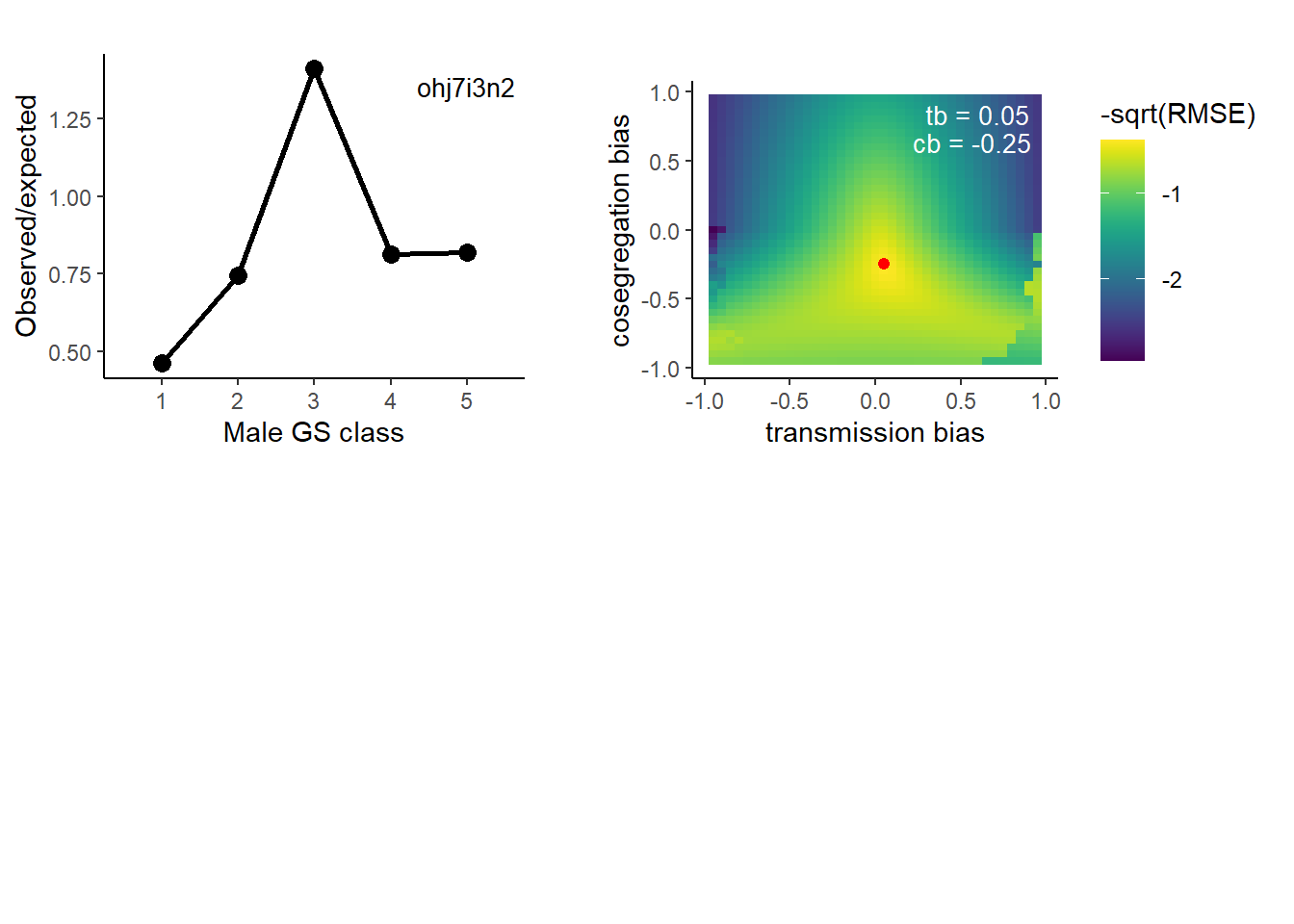

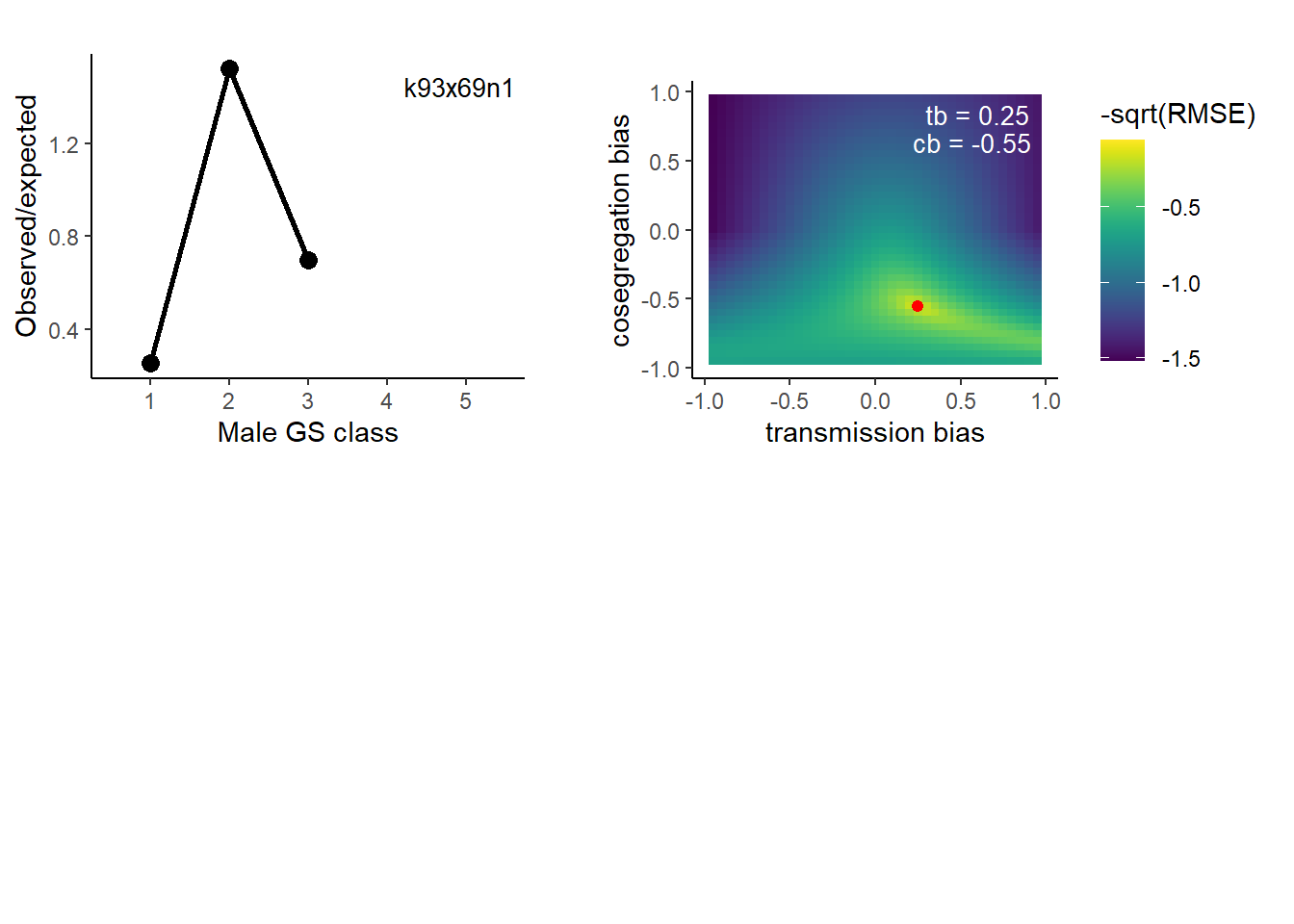

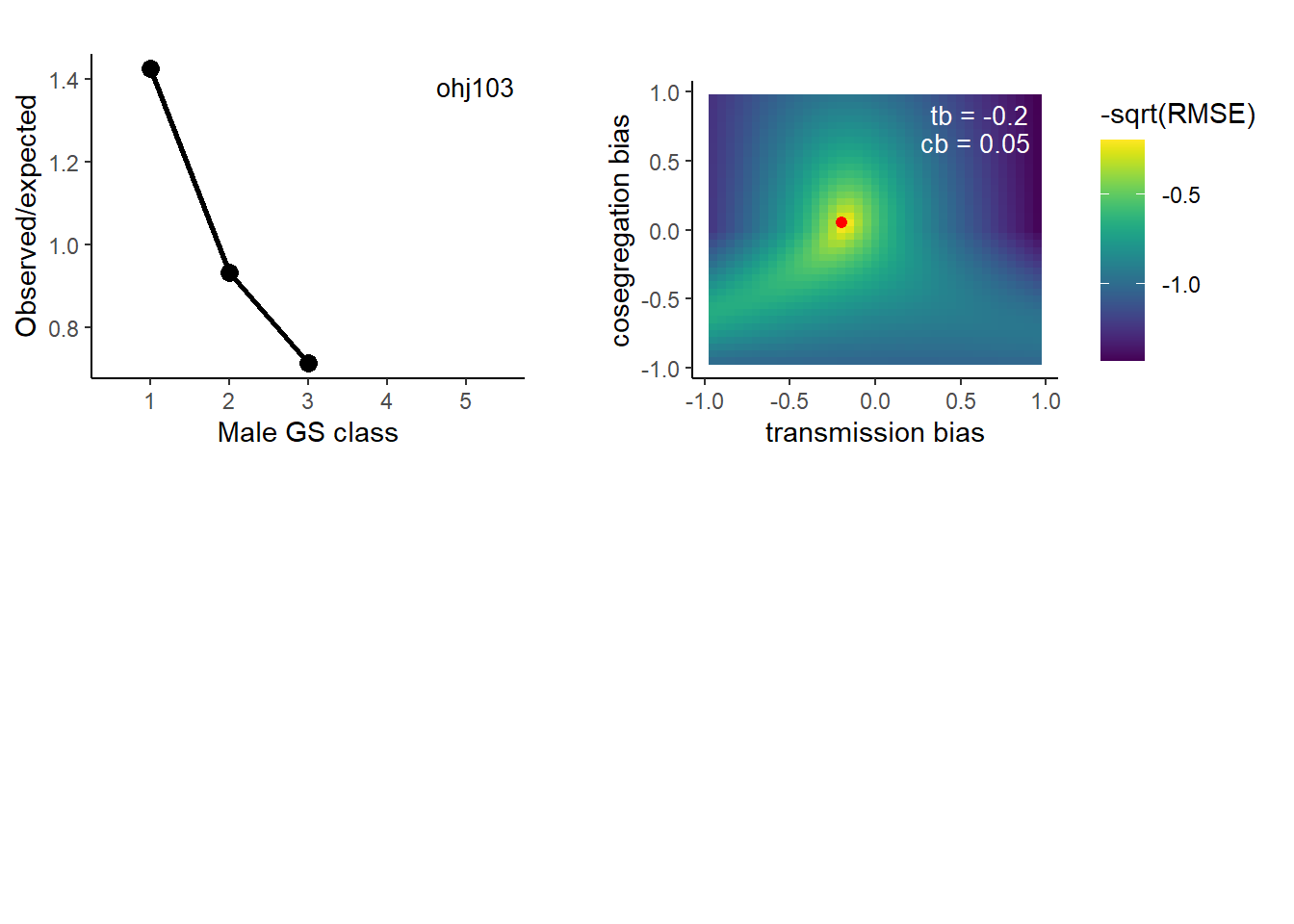

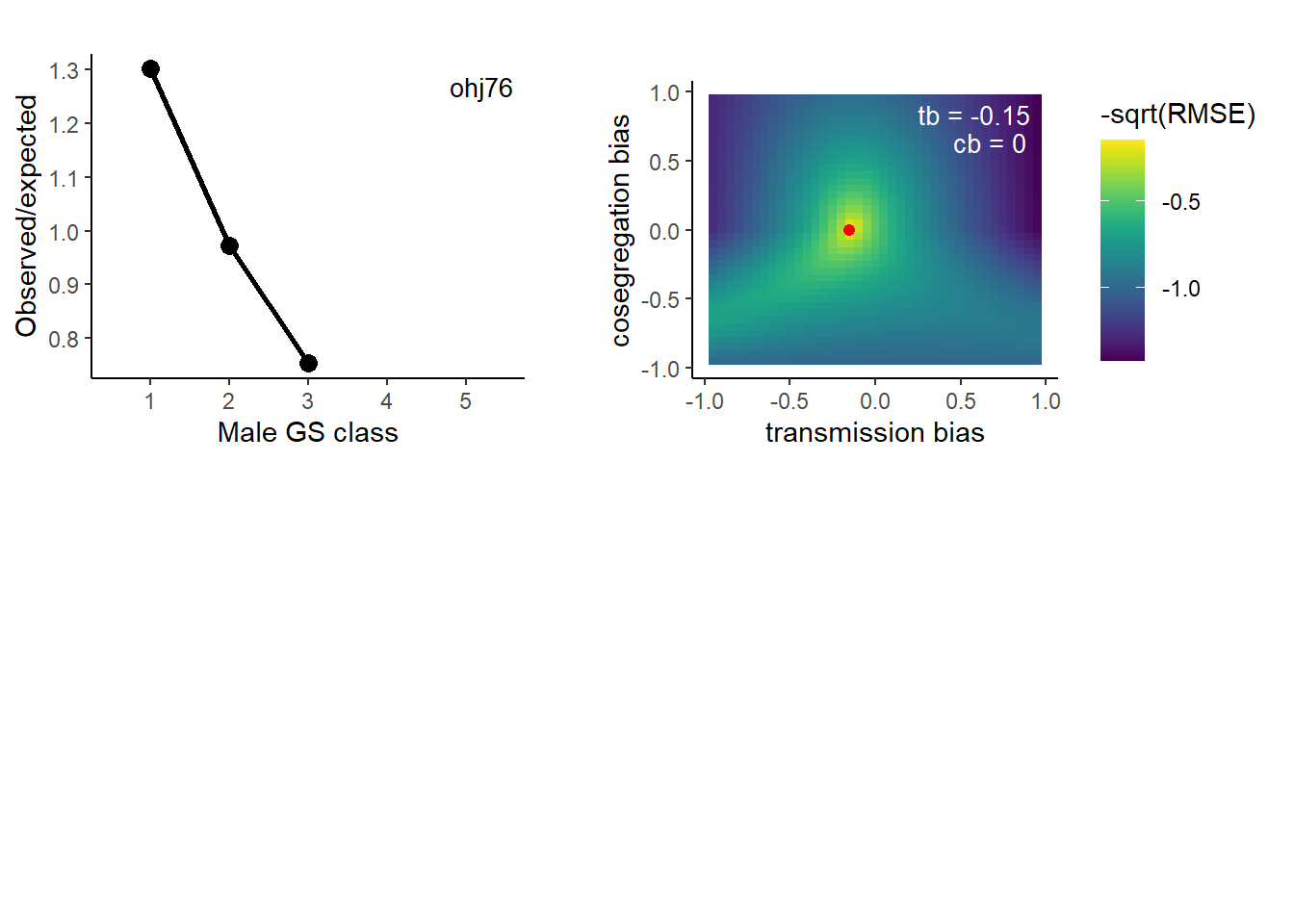

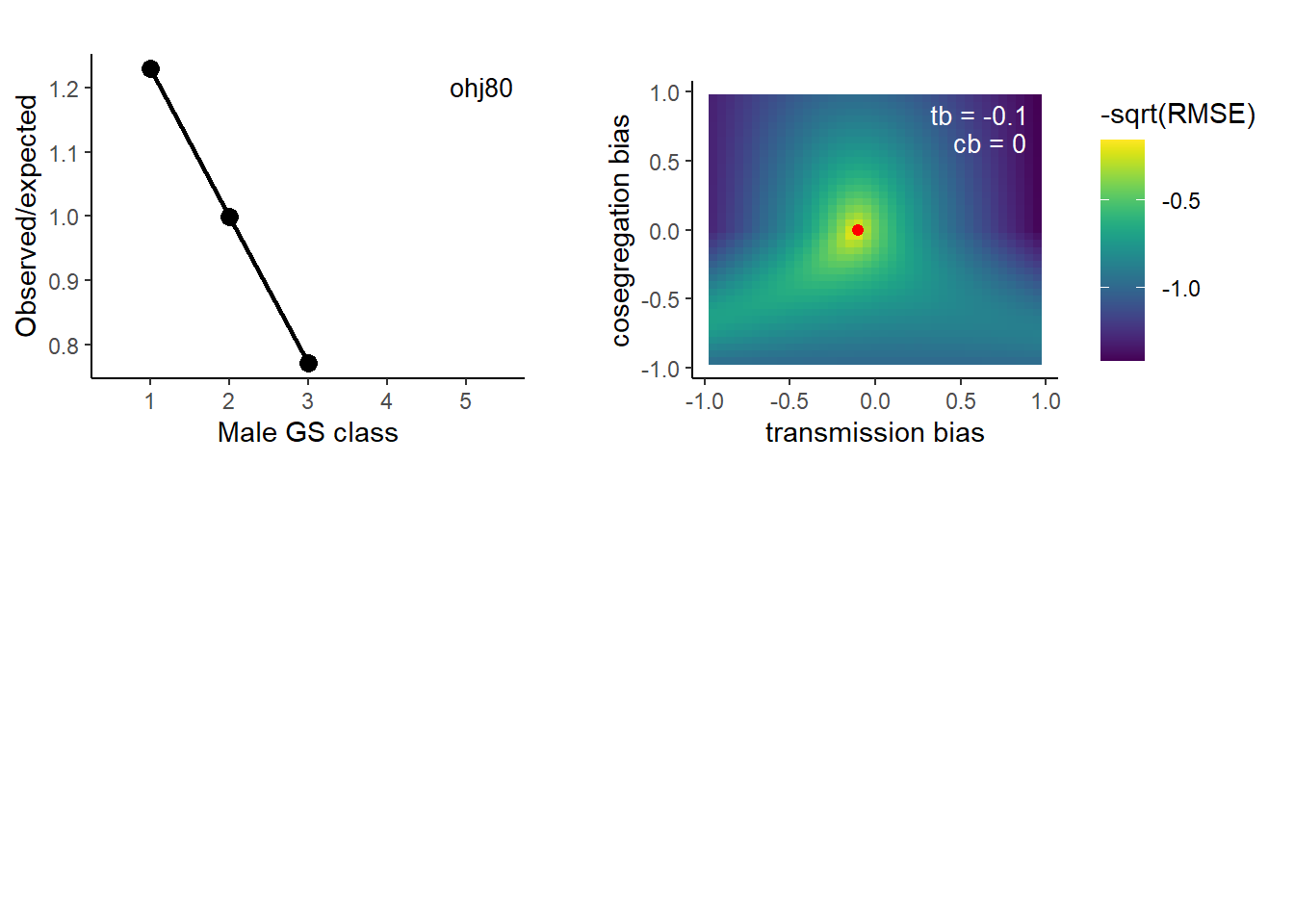

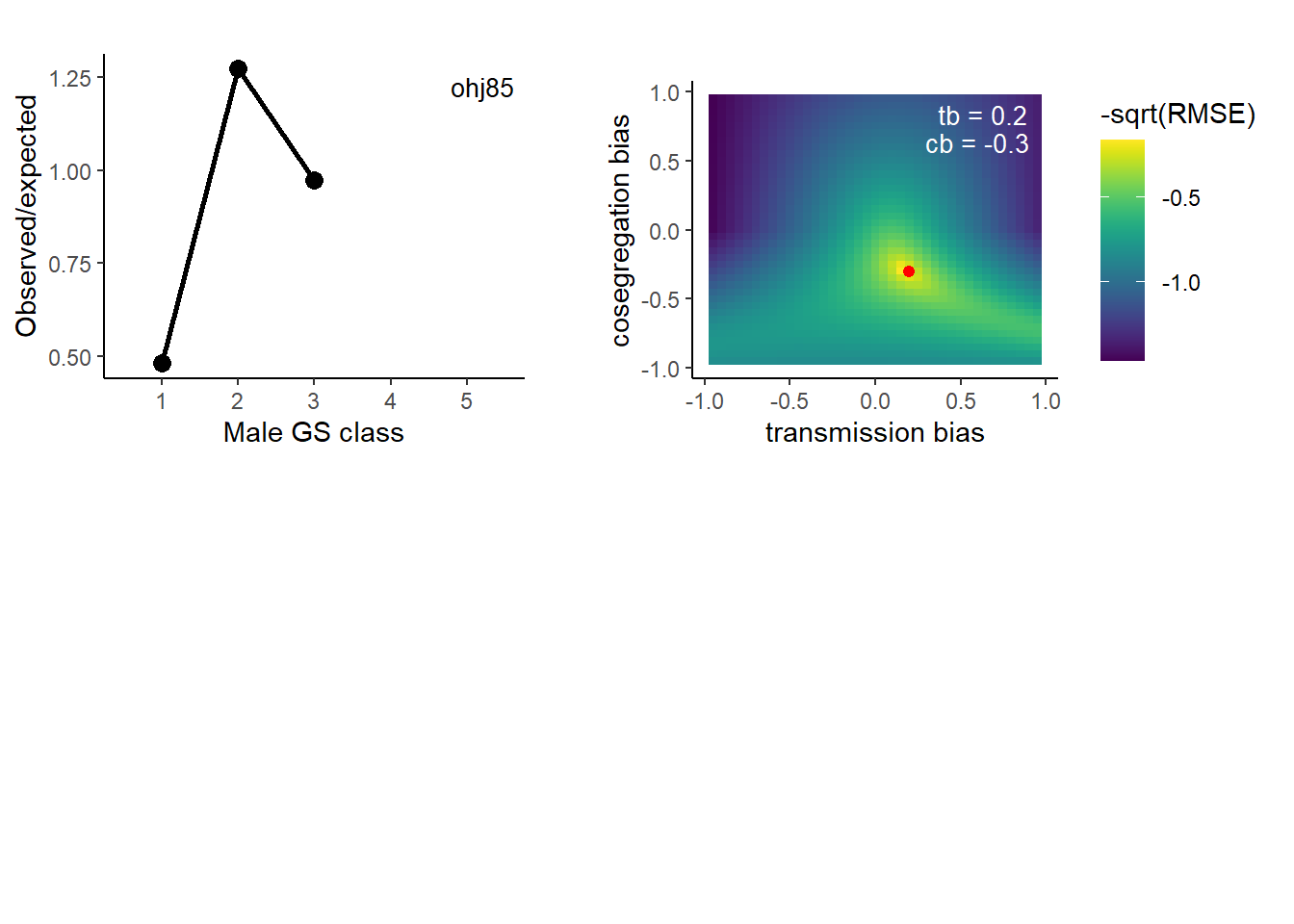

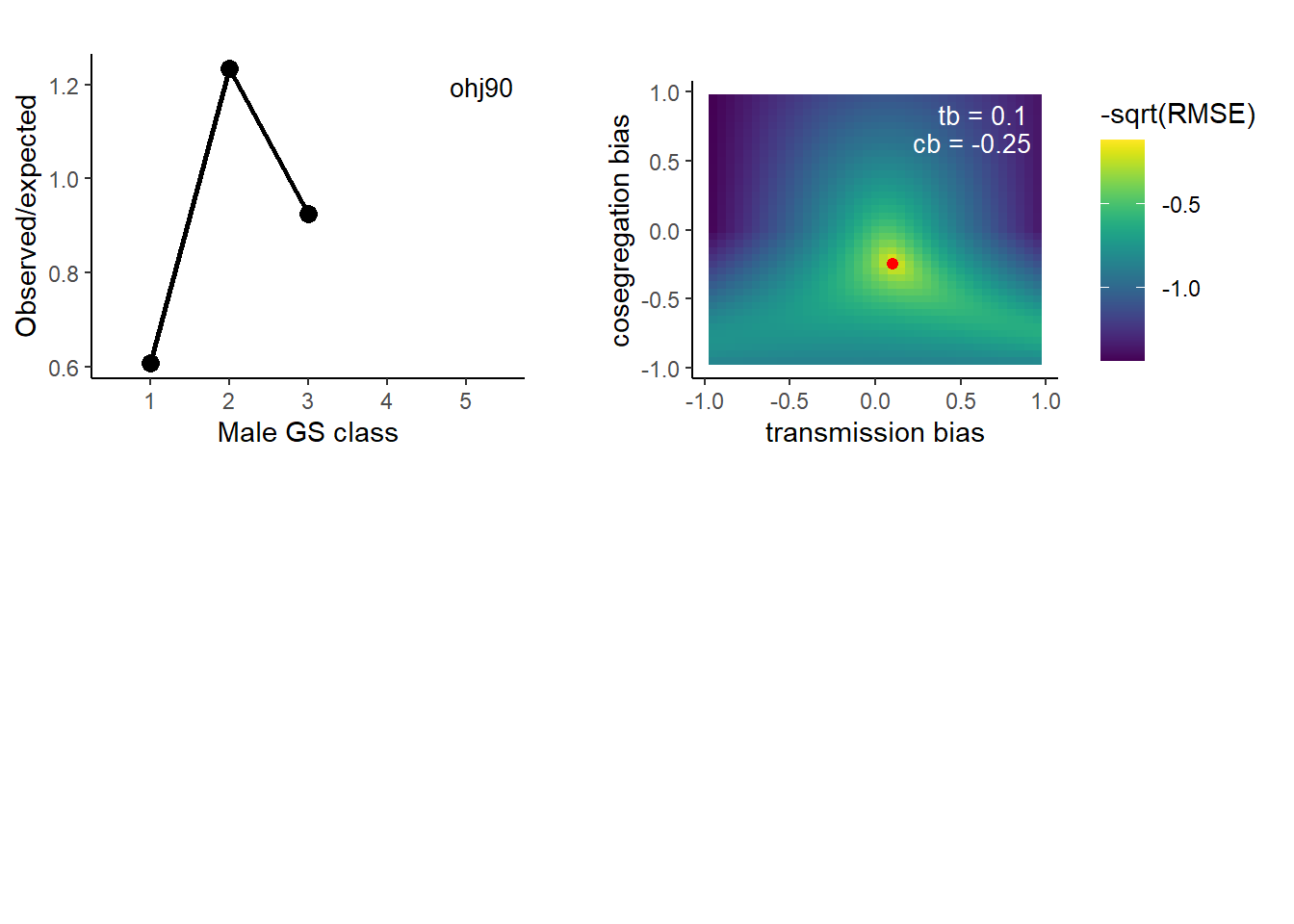

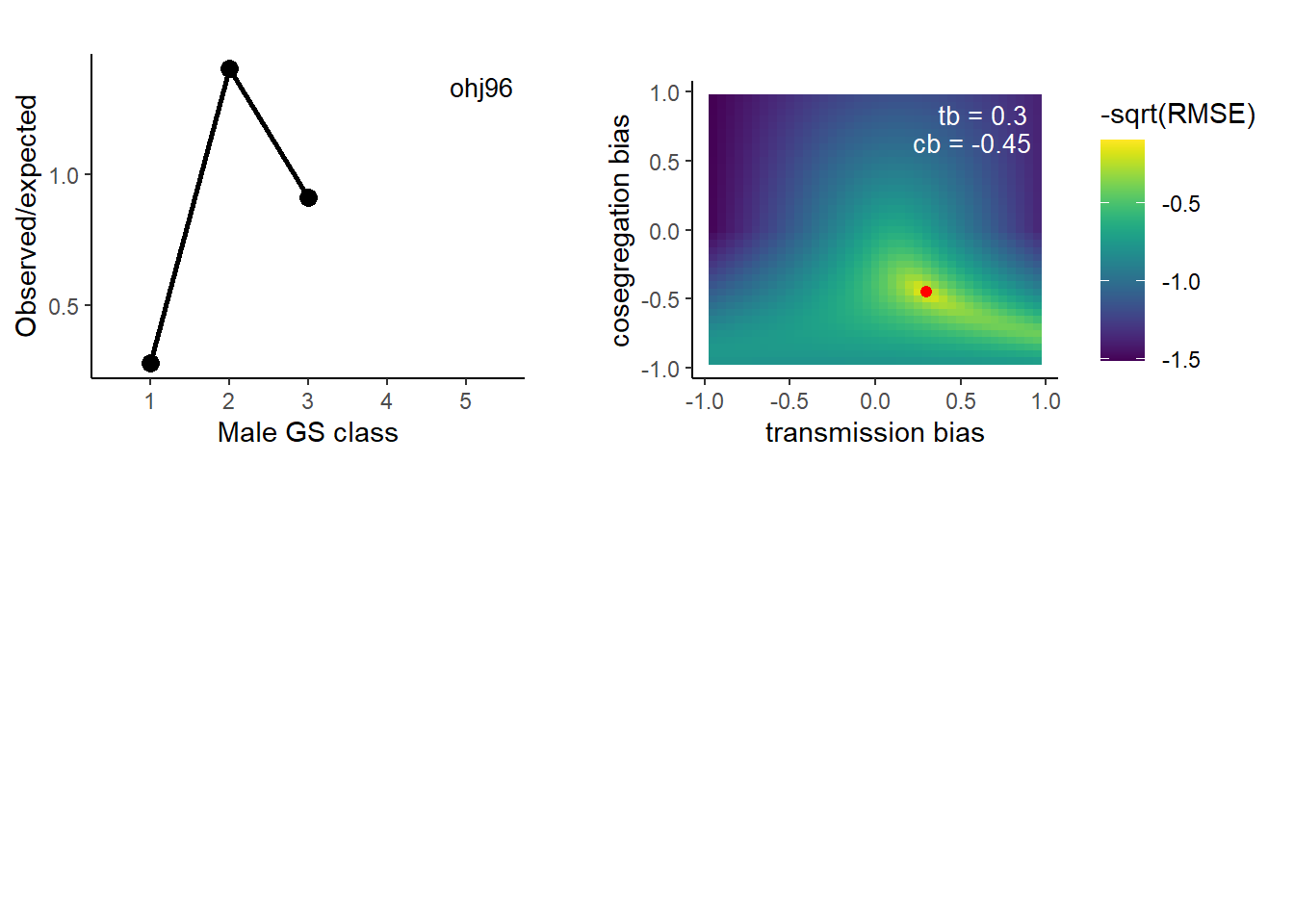

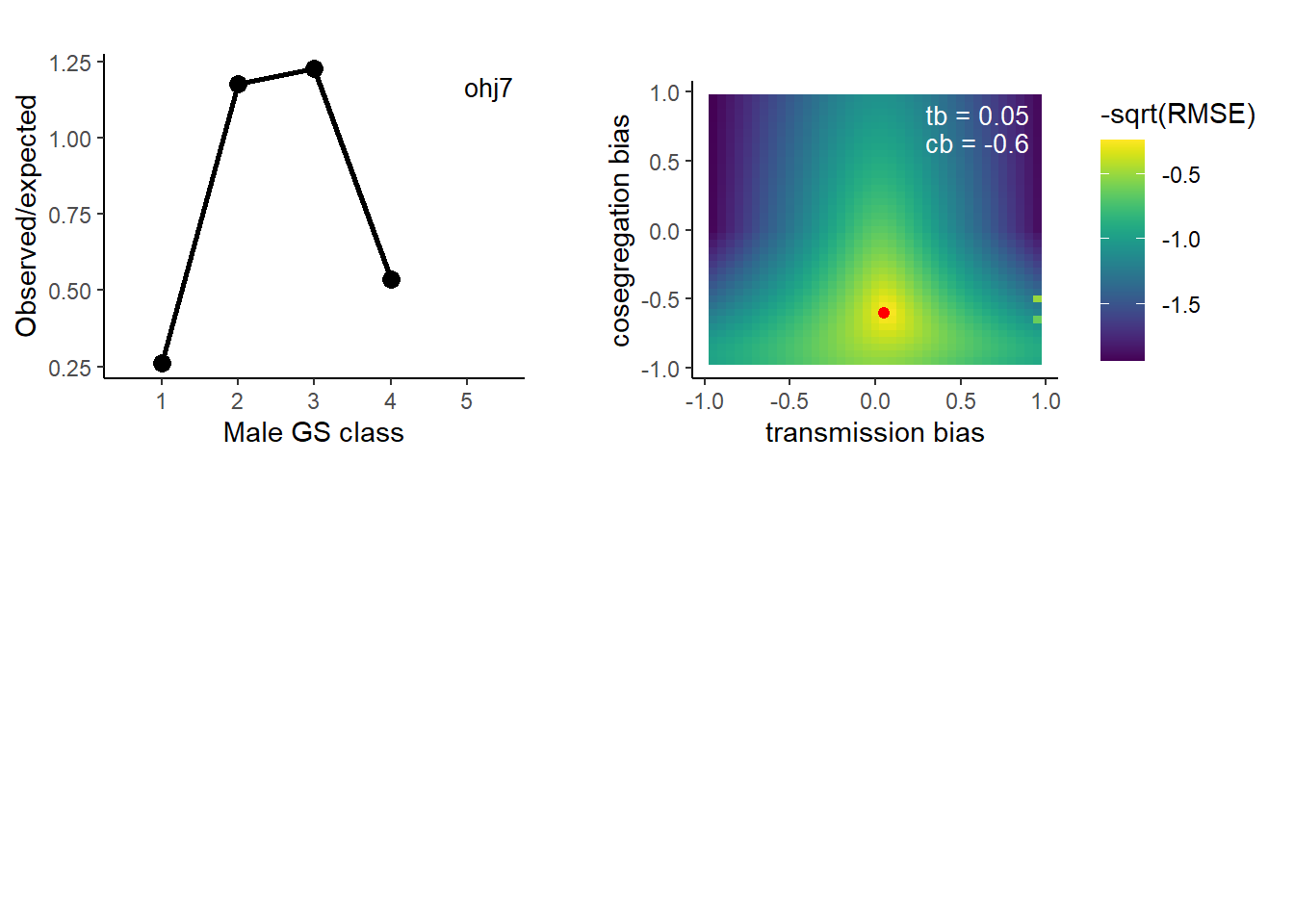

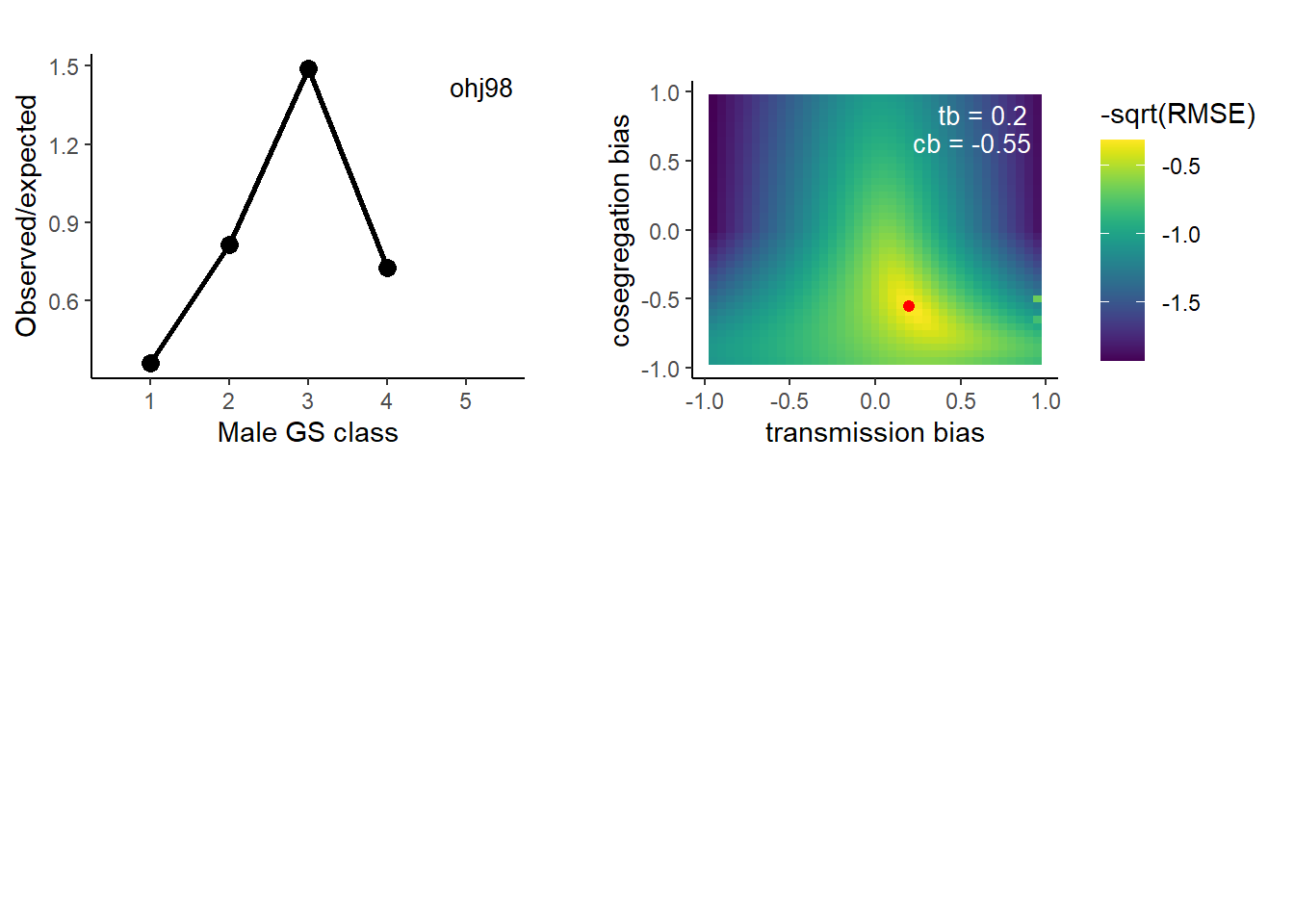

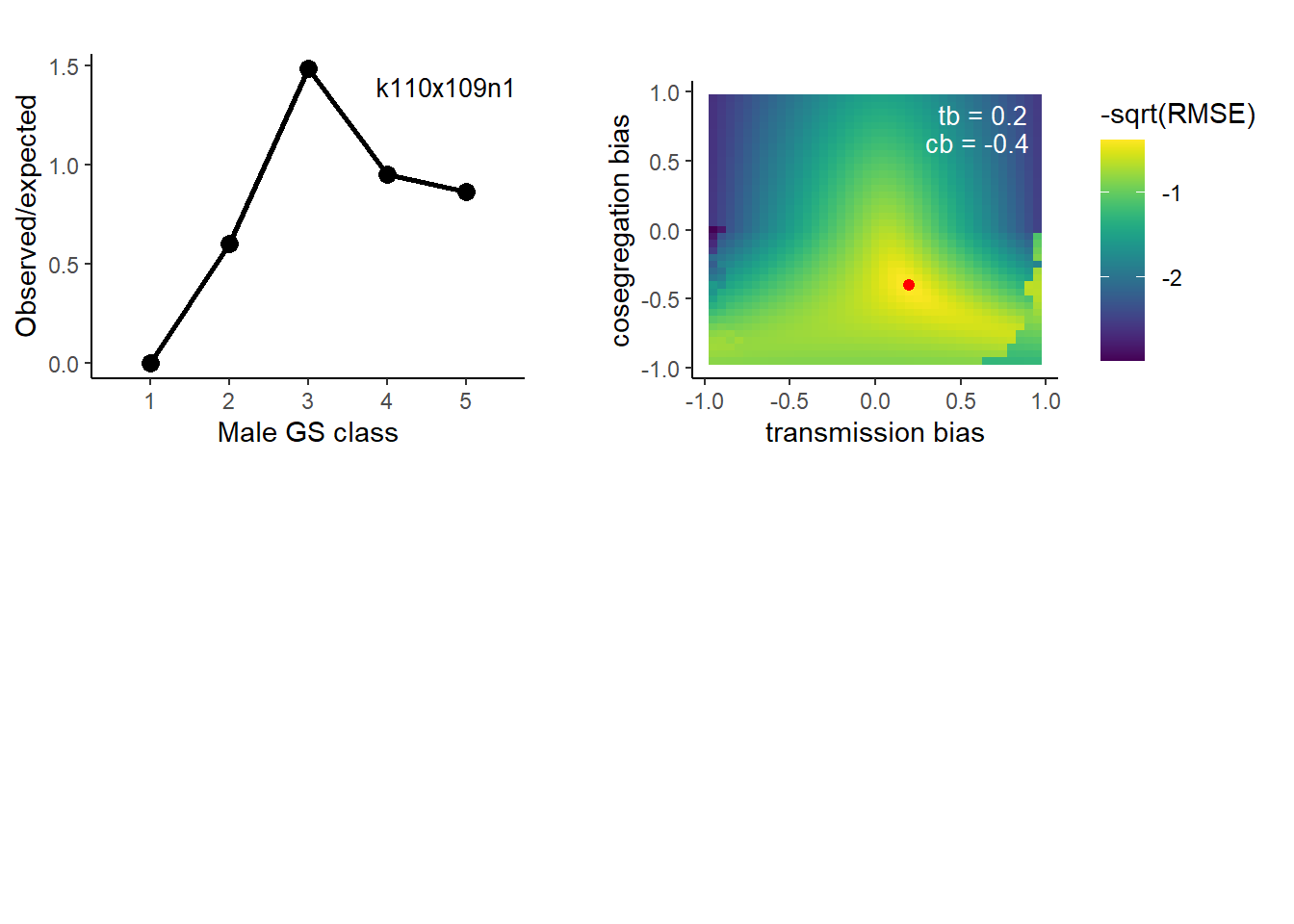

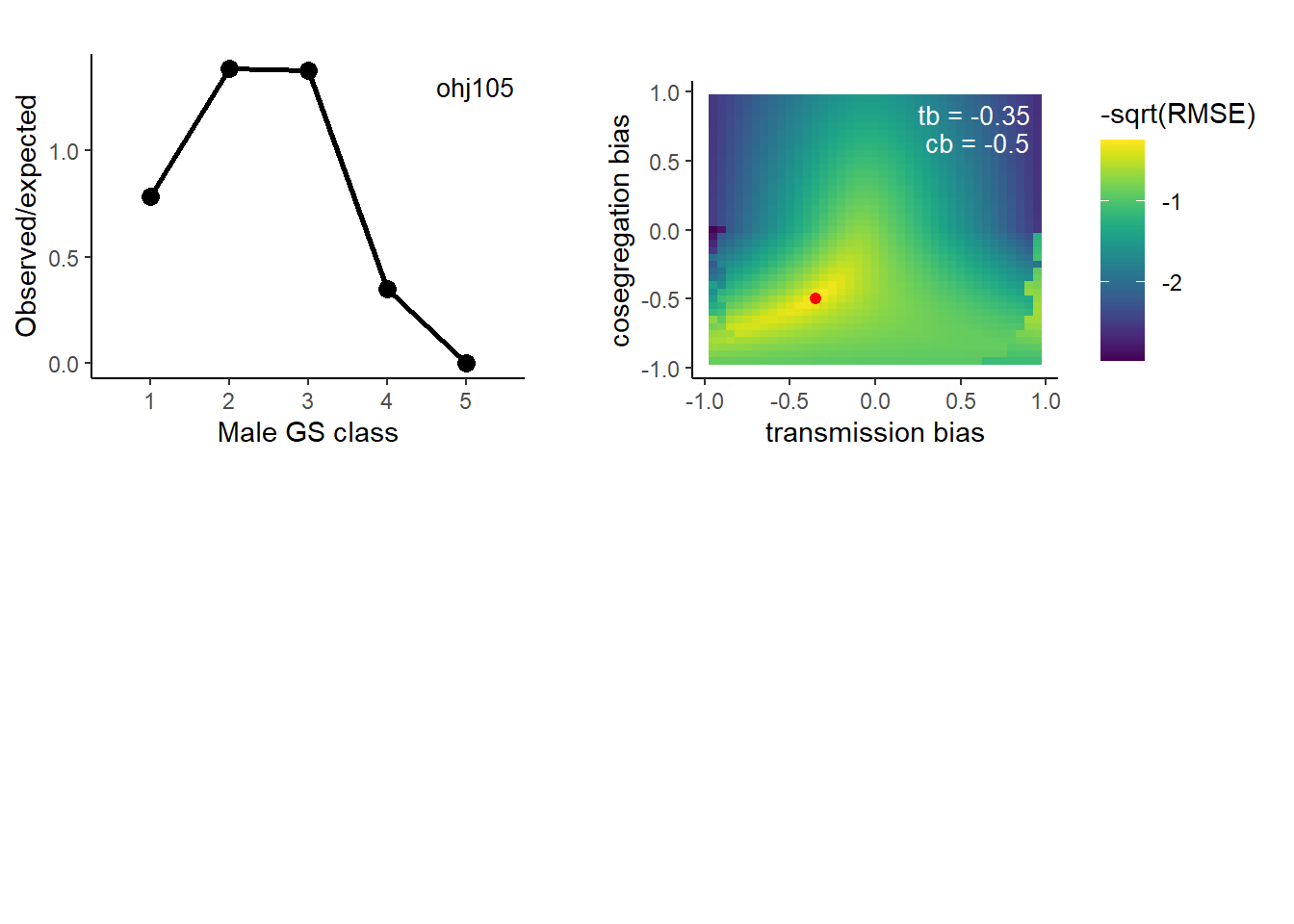

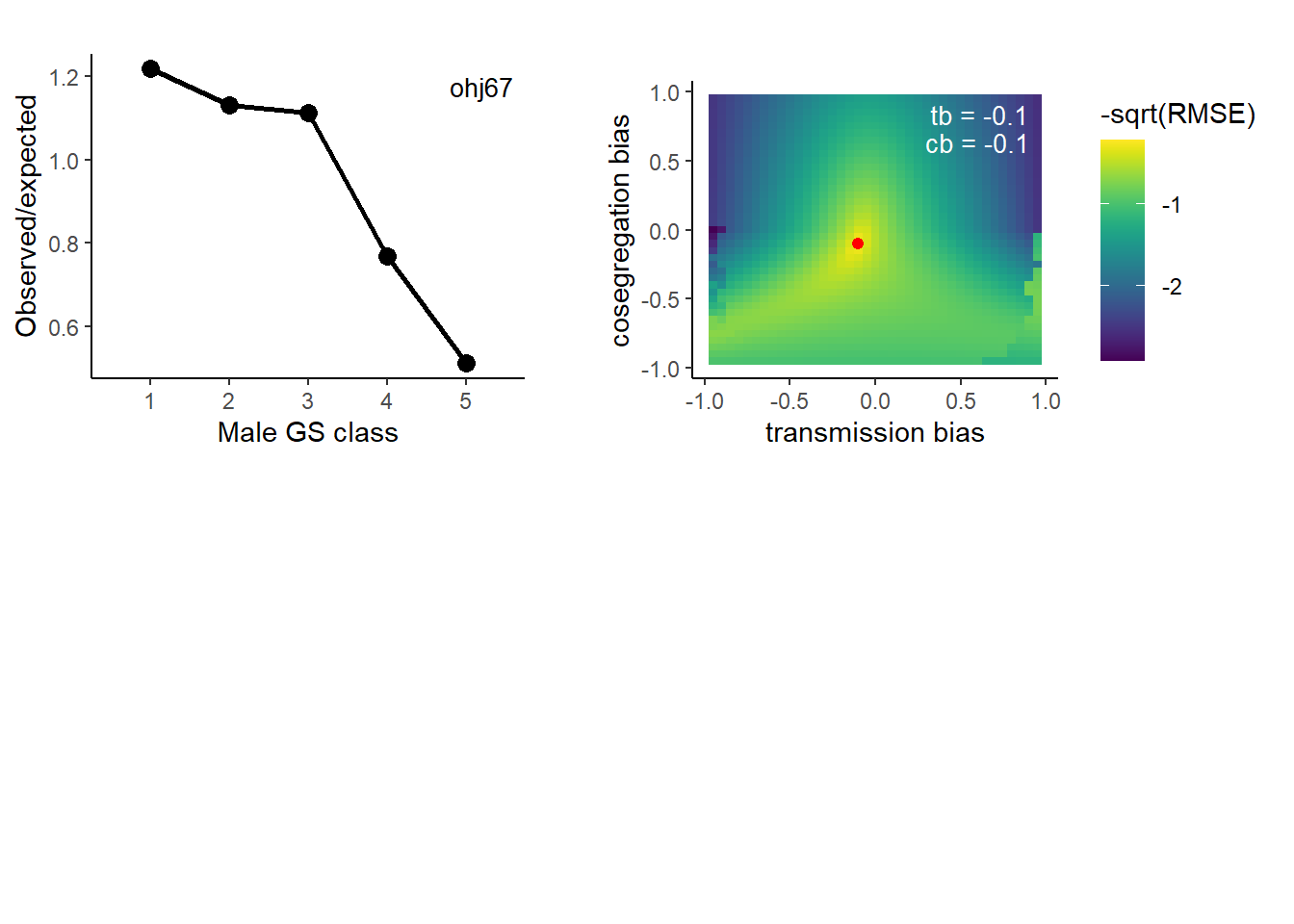

Supplement: Supplementary file 3 — Supplementary Information 3. [file 41598_2022_25566_MOESM3_ESM.docx]
